# A comprehensive multiomics approach toward understanding the relationship between aging and dementia

**DOI:** 10.18632/aging.100838

**Published:** 2015-11-11

**Authors:** Antonio Currais, Joshua Goldberg, Catherine Farrokhi, Max Chang, Marguerite Prior, Richard Dargusch, Daniel Daugherty, Aaron Armando, Oswald Quehenberger, Pamela Maher, David Schubert

**Affiliations:** ^1^ The Salk Institute for Biological Studies, La Jolla, CA 92037, USA; ^2^ Department of Medicine, University of California San Diego, CA 92093-0601, USA

**Keywords:** aging, Alzheimer's disease, multiomics, SAMP8 mice, inflammation, J147

## Abstract

Because age is the greatest risk factor for sporadic Alzheimer's disease (AD), phenotypic screens based upon old age-associated brain toxicities were used to develop the potent neurotrophic drug J147. Since certain aspects of aging may be primary cause of AD, we hypothesized that J147 would be effective against AD-associated pathology in rapidly aging SAMP8 mice and could be used to identify some of the molecular contributions of aging to AD. An inclusive and integrative multiomics approach was used to investigate protein and gene expression, metabolite levels, and cognition in old and young SAMP8 mice. J147 reduced cognitive deficits in old SAMP8 mice, while restoring multiple molecular markers associated with human AD, vascular pathology, impaired synaptic function, and inflammation to those approaching the young phenotype. The extensive assays used in this study identified a subset of molecular changes associated with aging that may be necessary for the development of AD.

## INTRODUCTION

There is currently no drug to prevent or slow down the progression of AD pathology. Our laboratory uses a drug discovery paradigm based upon a set of cell-based screening assays that mimic numerous aspects of old age-associated neurodegeneration and AD pathology [1]. This approach has led to the identification of J147, a very potent neuroprotective small molecule that is orally active in transgenic human familial AD (hFAD) animal models [2, 3].

Age is by far the greatest risk factor for dementia [4]. One model of aging is the senescence-accelerated prone 8 (SAMP8) mouse, that has a progressive, age-associated decline in brain function similar to human AD patients [5, 6]. As they age, SAMP8 mice develop an early deterioration in learning and memory as well as a number of pathophysiological alterations in the brain including increased oxidative stress, inflammation, vascular impairment, gliosis, Aβ accumulation and tau hyperphosphorylation. Therefore, the SAMP8 mice together with their response to J147 may help to delineate an understanding of the molecular mechanisms that are shared by aging and disease. These insights could lead to novel interventions for old age-associated sporadic AD.

To investigate the interaction between aging and the AD drug candidate J147 on brain function as well as brain and systemic metabolism, an integrative multi-omics approach was carried out in SAMP8 mice. Changes in behavior, protein expression, levels of metabolites and the whole transcriptome in old SAMP8 mice fed with control or J147 diets were compared with young SAMP8 control mice. These data identify a subset of metabolic changes associated with aging that may be relevant to sporadic AD and other forms of dementia. Importantly, the data demonstrate the ability of J147 to suppress many of these changes.

## RESULTS

To address the effect of J147 on the SAMP8 phenotype, two groups of three-month old mice were fed with control or J147 diet for an additional seven months, while another group of three-month old mice was used as a young control group. The SAMP8 mice are an inbred strain and, as such, young SAMP8 mice were chosen as controls for young age. Given the seven-month duration of the feeding paradigm, the effect of the J147 diet could only be assessed in old SAMP8 mice, and age-related changes were defined by comparison to the young SAMP8 animals. At 10 months of age, SAMP8 mice present strong age- and AD-associated brain deterioration [5–8]. The overall goal of this work was to use an in depth multiomics approach to identify molecules that mediate the physiological effects of J147 on the aging and AD-associated phenotypes of these mice.

### Behavioral assessment

**Figure 1:**
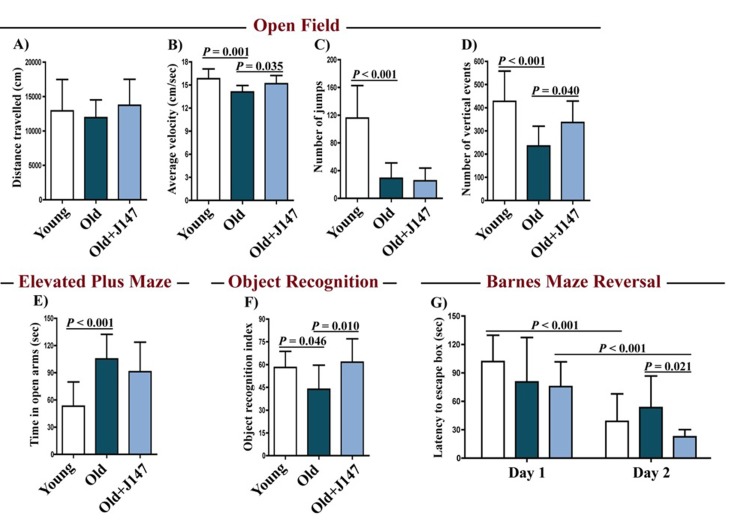
J147 improves locomotor and cognitive function in old SAMP8 mice Distance travelled (**A**), average velocity (**B**), number of jumps (**C**) and number of vertical events (**D**) were assessed in young mice and old SAMP8 mice fed with control or J147 diets with the open field test. The elevated plus maze (**E**) was used to measure anxiety levels. Recognition memory and spatial learning/memory were evaluated by the object recognition (**F**) and the Barnes maze (**G**) assays, respectively. One-way ANOVA followed by Tukey-Kramer post-hoc test and two-way repeated measures ANOVA and post hoc Bonferroni corrected t-test (n = 12-16/group). All data are mean ± SD.

By monitoring the spontaneous behavior of mice in the open field assay, we found a decline in activity parameters between the young and the old SAMP8 (Fig. 1 A–D). J147 had a positive effect on locomotor activity as it improved the average velocity and the number of vertical counts in the old SAMP8 mice. J147 had no effect on the body weights (Fig. S1).

To investigate whether J147 could prevent age-associated cognitive decline, mice were tested using the elevated plus maze (Fig. 1 E), the object recognition test (Fig. 1 F) and the Barnes maze (Fig. 1 G). The elevated plus maze examines disinhibition behavior based on the aversion of normal mice to open spaces. Dementia is clinically associated with disinhibition and AD mouse models tend to exhibit increased disinhibition [3]. Accordingly, old SAMP8 mice spent significantly more time in the open arms compared to the young SAMP8 mice (Fig. 1 E). However, this was not altered by J147 treatment.

The object recognition test evaluates recognition memory and is based on the spontaneous tendency of mice to spend more time exploring a novel object than a familiar one. The choice to explore the novel object reflects the use of learning and recognition memory. There was a significant decrease in the recognition index with age in SAMP8 mice, which was reversed by J147 (Fig. 1 F).

The Barnes maze is used to analyze spatial learning and hippocampal-dependent memory. In this assay, mice use visual cues to locate a hidden box. With repeated trials, animals with an intact memory show a significant reduction in the time (latency) to locate the box. If the box is moved to another location in the maze (reversal test), normal animals rapidly disengage from the previously learned information and re-learn the new location. No changes between the three groups were found in the escape latencies during the learning and the retention phases (data not shown). However, when tested during the reversal phase, which is more sensitive to smaller deficits in memory and learning, differences were found in the capacity of mice to relearn the new location of the escape box (Fig. 1 G). Importantly, J147 significantly improved learning of the new location. Altogether, these data show that J147 prevents the deterioration of several aspects of behavior and memory that are altered in old SAMP8 mice.

### Brain hippocampal protein expression

**Figure 2:**
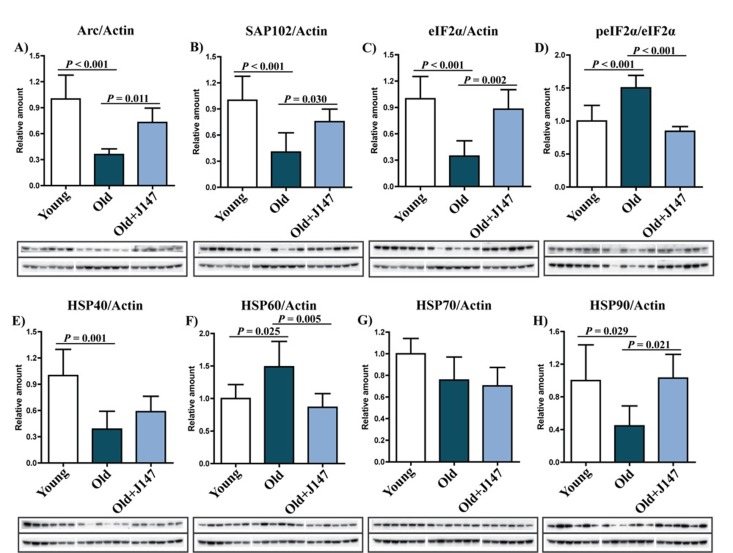
Dysregulation of neuronal homeostasis and stress responses in the hippocampus of old SAMP8 mice is partially restored by J147 RIPA-soluble fractions from hippocampal tissue were analyzed by Western blotting for relevant markers of neuronal homeostasis and stress and are presented relative to actin or the unphosphorylated molecule: Arc (**A**), SAP102 (**B**), eIF2α (**C**), peIF2α (**D**), HSP40 (**E**), HSP60 (**F**), HSP70 (**G**), HSP90 (**H**). One-way ANOVA followed by Tukey-Kramer post-hoc test (n = 6/group). All data are mean ± SD.

Western blotting was used to investigate both protein alterations underlying the decline in cognitive performance of old SAMP8 mice and the therapeutic effects of J147. The expression of activity-regulated cytoskeleton-associated protein (Arc) and synapse-associated protein 102 (SAP102) decreased in old mice compared to young mice (Fig. 2 A and B), and treatment with J147 prevented these decreases. It was then asked if these changes were accompanied by alterations in the levels of proteins involved in the cellular responses to stress relevant to aging and AD (Fig. 2 C–H). Phosphorylation of eukaryotic initiation factor 2a (eIF2α) occurs under a variety of stress conditions to control protein synthesis. Although total levels of eIF2α were decreased in old SAMP8 mice compared to young controls, its phosphorylation was increased (Fig. 2 C and D), as has been reported in AD patients [9]. Importantly, J147 reverted the changes in both the total levels and the phosphorylation of eIF2α. Additionally, while the levels of heat shock protein 70 (HSP70) were not significantly altered between groups, changes in HSP40, HSP60 and HSP90 were detected in the old SAMP8 mice (Fig. 2 E–H). J147 restored HSP60 and HSP90 to levels similar to those found in the young control mice.

**Figure 3:**
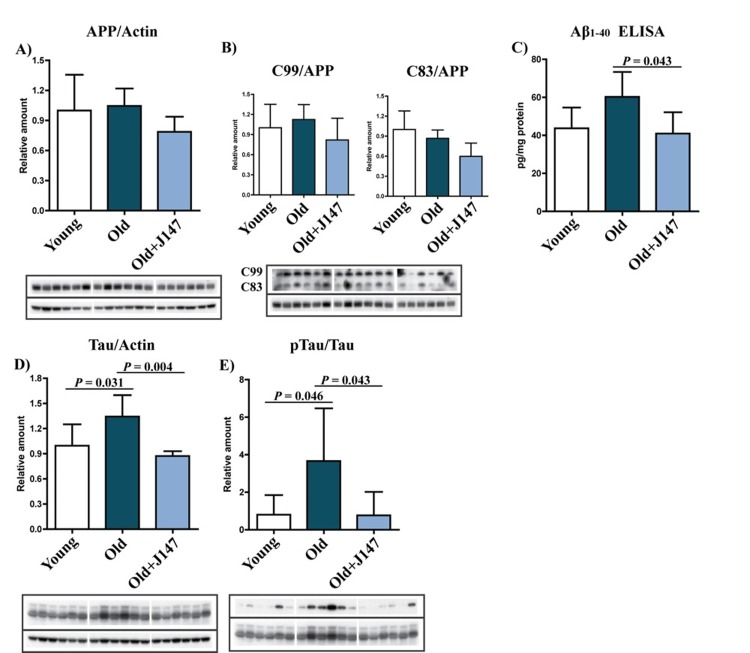
J147 prevents alterations in Aβ and tau homeostasis in the hippocampus of old SAMP8 mice (**A** and **B**) Western blot analysis of APP processing in hippocampal tissue using an antibody against the C-terminus of APP. Full-length APP and the APP cleavage products C99 and C83 were detected. (**C**) ELISA for Aβ. Western blot analysis of total Tau (**D**) and pTau Ser396 (**E**). One-way ANOVA followed by Tukey-Kramer post-hoc test (n = 6/group). All data are mean ± SD.

One hallmark of the AD brain is extracellular Aβ plaques. Although SAMP8 mice do not develop classical plaque pathology, they have a high content of Aβ and amyloid deposition around blood vessels [5, 7]. Aβ is the product of sequential cleavages of the amyloid precursor protein (APP). Processing of APP involves the formation of the C83 and C99 C-terminal fragments by α and β-secretases, respectively. Amyloidogenic processing of C99 by γ-secretase then generates Aβ [10]. Although no significant changes in the total levels of APP and the C99 and C83 fragments across the three groups were identified, there was a trend towards lower levels of APP and both the C99 and C83 fragments after treatment with J147 (Fig. 3 A and B). More importantly, an increase in the level of Aβ_1-40_ was detected in the hippocampus of old SAMP8 mice, which was significantly prevented by J147 (Fig. 3 C). Aβ_1-42_ was below the limit of detection (data not shown).

Tau pathology is another important feature of AD. Old SAMP8 mice showed increases in both tau protein and its phosphorylation at Ser396 (Fig. 3 D and E), an epitope affected in the human AD brain [11]. J147 prevented both of these alterations.

### Vascular dysfunction and inflammation

**Figure 4:**
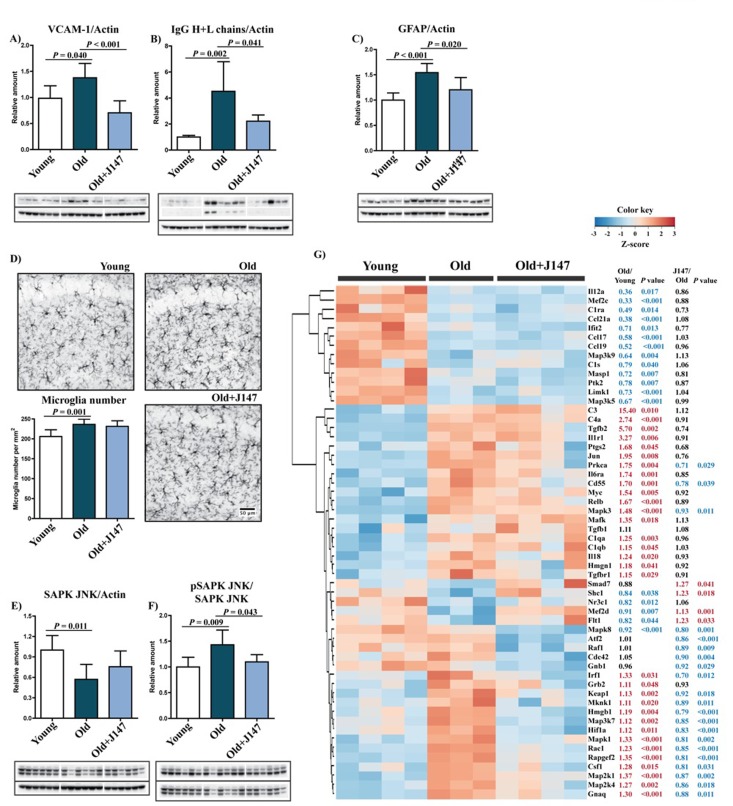
Increased inflammation and gliosis in the hippocampus of old SAMP8 mice are prevented by J147 Western blot analysis of the marker for vascular endothelial inflammation VCAM-1 (**A**) and of the IgG (Heavy + Light chains) content (**B**). (**C**) Astrocytosis, measured by Western blot of GFAP levels. One-way ANOVA followed by Tukey-Kramer post-hoc test (n = 6/group). (**D**) Microgliosis was assessed by immunohistochemical (IHC) staining and number of Iba-1-positive cells per mm^2^ of total hippocampus calculated. Original magnification: x100. One-way ANOVA followed by Tukey-Kramer post-hoc test (n = 8/group). (**E** and **F**) Activation of the stress/inflammation-associated SAPK/JNK was measured by Western blot analysis of its phosphorylation at Thr183/Tyr185. One-way ANOVA followed by Tukey-Kramer post-hoc test (n = 6/group). All data are mean ± SD. (**G**) Quantitative RNA analysis of altered genes related to inflammation. Heatmap and hierarchical clustering of scaled gene expression with respective fold changes and *P* values for the comparisons Old/Young and Old+J147/Old. Scaled expression value (Z-score) is plotted in red-blue color scale with red indicating high expression and blue indicating low expression. One-way ANOVA followed by Tukey-Kramer post-hoc test (n = 3-4/group).

Given the relevance of inflammation in aging and AD, a detailed characterization of the inflammatory status of the aged SAMP8 brain and of the effects of J147 was carried out. AD is often accompanied by inflammation of the brain blood barrier (BBB), and the disruption of its permeability severely compromises neuronal homeostasis [12, 13]. A significant increase in the levels of vascular cell adhesion molecule 1 (VCAM-1), a protein associated with vascular endothelium inflammation, was detected in the hippocampus of old SAMP8 mice compared to the young SAMP8 controls (Fig. 4 A). This increase was completely prevented by J147 treatment. In addition, old mice showed significantly higher levels of endogenous immunoglobulin G (IgG) (Fig. 4 B), a consequence of disrupted BBB permeability [14], which was also prevented by J147. Together these results suggest that J147 helps to preserve BBB homeostasis and vascular function in aged SAMP8 mice.

Astrocytes are key constituents of the BBB, and astrocytic reactivity is increased in AD [15]. We previously reported an increased expression of glial fibrillary acidic protein (GFAP), a marker for astrocytes, due to an increased number of astrocytes in the hippocampus of old SAMP8 mice [7]. The current study confirmed an increase in GFAP levels from young to old SAMP8 mice and showed that J147 reduced GFAP expression (Fig. 4 C). The number of microglia increased in the hippocampus of old mice compared to young (Fig. 4 D), but J147 did not significantly alter their number.

Western blot analysis revealed an activation of the stress-activated protein kinase/Jun-amino-terminal kinase (SAPK/JNK), determined by its phosphorylation, in the hippocampus of old SAMP8 mice (Fig. 4 E and F). SAPK/JNK is activated in AD brains and may be the cause of abnormal tau phosphorylation [16]. Importantly, J147 prevented the activation of SAPK/JNK in the old mice.

To expand upon these findings, the expression of a comprehensive panel of inflammatory genes was analyzed. The expression of a large number of genes was altered between young and old SAMP8 mice (50/248); most were upregulated (Fig. 4 G). The vast majority of changes associated with J147 reverted alterations found in old SAMP8 mice toward expression levels in young mice. These include mitogen-activated protein kinase (MAPK) kinases, such as Map3k7 and Map2k4, that are direct activators of SAPK/JNK, in accordance with the Western blotting data (Fig. 4 E and F). J147 treatment was largely associated with an overall decrease in the expression of inflammatory markers in old mice, indicative of a reduction in stress-associated inflammation. However, the expression of some genes that are elevated in old mice, such as the components of the complement system C1qa, C1qb, C3 and C4a, were not altered by J147. Interestingly, there was a group of inflammation-associated genes whose expression was lowered in the old mice. Although most of these were not changed by J147, one that was restored by J147 is *Flt1* that encodes the vascular endothelial growth factor receptor 1 and may be related to the effect of J147 on the brain vasculature. These data show that J147 prevents a portion of the pro-inflammatory changes associated with aging in the SAMP8 mice.

### Eicosanoid metabolism

**Figure 5:**
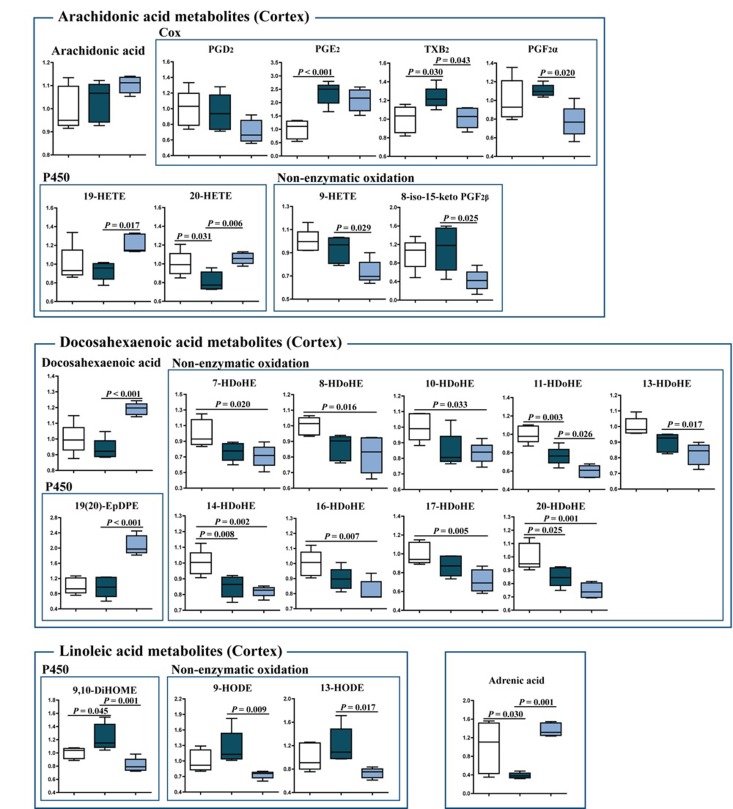
Changes in eicosanoid metabolism of fatty acids in the cortex of young SAMP8, old SAMP8 and old SAMP8 mice fed with J147 Significant changes in the metabolites of arachidonic acid, docosahexaenoic acid, linoleic acid and adrenic acid derived from the actions of COX and cytochrome P450 and non-enzymatic oxidation. One-way ANOVA followed by Tukey-Kramer post-hoc test (n = 5/group). Values are expressed as box-and-whisker plots.

To further elucidate the effects of J147 on inflammation, a detailed analysis of eicosanoid production in the brain cortex was conducted. Eicosanoids are a class of bioactive lipid mediators derived from the metabolism of polyunsaturated fatty acids (PUFAs) by cyclooxygenases (COXs), lipoxygenases (LOXs) and cytochrome P450s as well as nonenzymatic pathways [17]. They are potent regulators of the inflammatory response in the periphery, but are much less studied in the brain. Several fatty acids, including arachidonic acid (AA), docosahexaenoic acid (DHA), linoleic acid (LA) and adrenic acid, as well as their respective metabolites were analyzed (Fig. 5). J147 significantly increased the levels of DHA and restored those of adrenic acid. J147 also had a strong anti-oxidant effect, since most of the metabolites derived from the non-enzymatic oxidation of the different fatty acids were decreased in mice treated with J147 as compared to either young or old SAMP8 mice. These include the AA-meta-bolites 9-HETE and 8-iso-15-keto PGF2β; the DHA-metabolites 11- and 13-HDoHEs; and the LA-metabolites 9-HODE and 13-HODE. Therefore, J147 may reduce the pro-oxidant status in the brain of old animals.

The levels of the cytochrome P450 metabolites 19-HETE and 20-HETE (AA derivatives), 19(20)-EpDPE (DHA derivative) and 9,10-DiHOME (LA derivative), which are known regulators of vascular dynamics [18, 19], were altered with J147 treatment. 20-HETE and 9,10-DiHOME were also altered by aging and this was prevented by J147. In addition, several COX metabolites were changed. TXB2, a product of TXA2, was increased in old SAMP8 mice and lowered by J147. The thromboxane pathway is implicated in platelet aggregation, adhesion and vascular contraction during inflammation [20]. Thus, these data further support the idea that J147 reduces the decline in brain vascular health that occurs during aging.

### Small molecule metabolism

**Figure 6:**
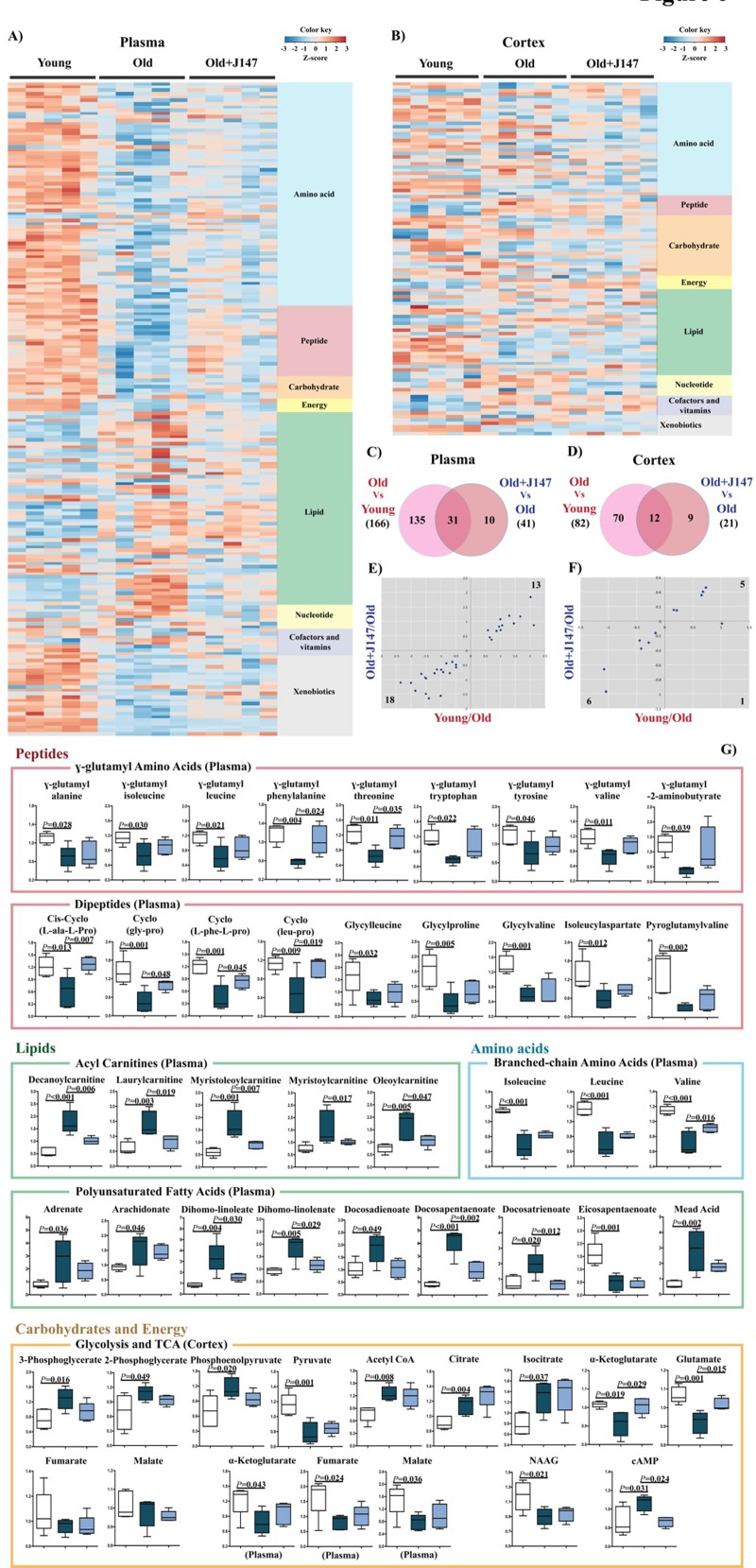
Metabolomic analysis of plasma and cortex demonstrate that alterations in biological pathways between young SAMP8 and old SAMP8 mice are partially rescued by J147 Plasma (**A**) and cortex (**B**) heatmaps of the biochemicals found significantly modified, organized by major biological groups. Scaled expression value (Z-score) is plotted in red-blue color scale with red indicating high expression and blue indicating low expression. Venn diagrams illustrating shared and uniquely affected metabolites in plasma (**C**) and cortex (**D**). Correlation of metabolite levels altered in Young/Old and Old+J147/Old in plasma (**E**) and cortex (**F**) (units are -log(fold change)). (**G**) Selection of relevant biochemicals changed between young and old SAMP8 mice affected by J147, including γ-glutamyl amino acids, dipeptides, BCAAs, acyl carnitines and PUFAs in the plasma, and metabolites related to neurotransmission and energetic pathways in the cortex. One-way ANOVA followed by Tukey-Kramer post-hoc test (n = 5/group). Values are expressed as box-and-whisker plots.

To address the possible therapeutic effects of J147 on brain and whole body health, a global metabolic profiling study was carried out with blood plasma and brain cortical tissue. 195 of 593 (32.9%) and 105 of 493 (21.3%) assayed biochemicals differed significantly in the plasma and cortex, respectively, between the three groups. The heatmaps in Figures 6 A and B depict the major altered biological pathways. Global pathway changes regarding the metabolism of amino acids, peptides and lipids were detected in the plasma (Fig. 6 A). In the brain, significant changes were associated with amino acid and lipid metabolism and, importantly, neurotransmission and energy production (Fig. 6 B).

Venn diagrams highlighting the significant changes in plasma and cortex that differentiate the comparisons between the young, old and old+J147 groups are shown in Figures 6 C and D. Fold changes of the overlapping metabolites were correlated between the two comparisons (Fig. 6 E and F). Treatment with J147 rescued changes in all of the 31 plasma metabolites also found altered in old SAMP8 mice. This accounts for 76% of all differences between old SAMP8 treated with J147 and old SAMP8 fed with control diet. In the cortex, J147 rescued changes in 11 biochemicals (out of 12), representing 55% of all differences between J147 treated and untreated old SAMP8 mice. Figure 6 G shows the specific biological groups of metabolites found affected in the plasma (for all biochemicals see Table S1). Numerous γ-glutamyl amino acids, branched-chain amino acids (BCAAs) and dipeptides were downregulated with age, while several acylcarnitines and PUFAs were highly elevated in the old SAMP8 mice. In both cases, J147 reversed most of these changes towards the younger phenotype.

To investigate which biological pathways and diseases/functions could be associated with these alterations, Ingenuity Pathways Analysis (IPA) was carried out with biochemicals present in the Human Metabolome Database (HMDB). It is important to note that many of the metabolites identified in Figure 6 do not have an established biological pathway in HMDB; therefore, this analysis has limitations. The top canonical pathways altered in the plasma are shown in Figure S2 A and B, and confirm changes in amino acid, protein metabolism, urea cycle and mitochondrial energetics (tricarboxycylic acid cycle (TCA) and oxidative phosphorylation) found between the young and old SAMP8 mice. Some of the diseases and functions predicted to be associated with these changes relevant to aging include cancer, gastrointestinal and hepatic dysfunction, endocrine system, energy metabolism and inflammatory responses (Fig. S2 C). Most of these were also found associated with the metabolites changed by J147, indicative that J147 might be rescuing certain aspects of these diseases and functions that are changed with age.

Although the total number of metabolites altered in the brain of old SAMP8 mice was lower than in the plasma, the associations determined by IPA were strong (Fig. S2 E and F). Some of the metabolites of interest participate in energy production processes, amino acid metabolism, G protein-coupled receptor (GPCR) and cAMP signaling, neuronal homeostasis and lipid metabolism (Fig. S2 E) (for all biochemicals see Table S2). Specifically, alterations in glycolytic and TCA intermediates in old SAMP8 mice (Fig. 6 G) are indicative of mitochondrial dysfunction, which is characteristic of aging and AD [21]. J147 also preserved the levels of glutamate, the principal neurotransmitter in the brain and a product of the TCA intermediate α-ketoglutarate, which was also rescued by J147 (Fig. 6 G). The levels of cAMP were elevated in old SAMP8 mice and were lowered by J147. cAMP is an intracellular signal transduction molecule crucial for many biological processes and its upregulation has been associated with AD [22]. The predicted diseases and functions are consistent with these alterations and include cancer, stress pathways, cellular survival/growth and maintenance, neurological disease and energy metabolism (Fig. S2 F).

### Whole transcriptome

**Figure 7:**
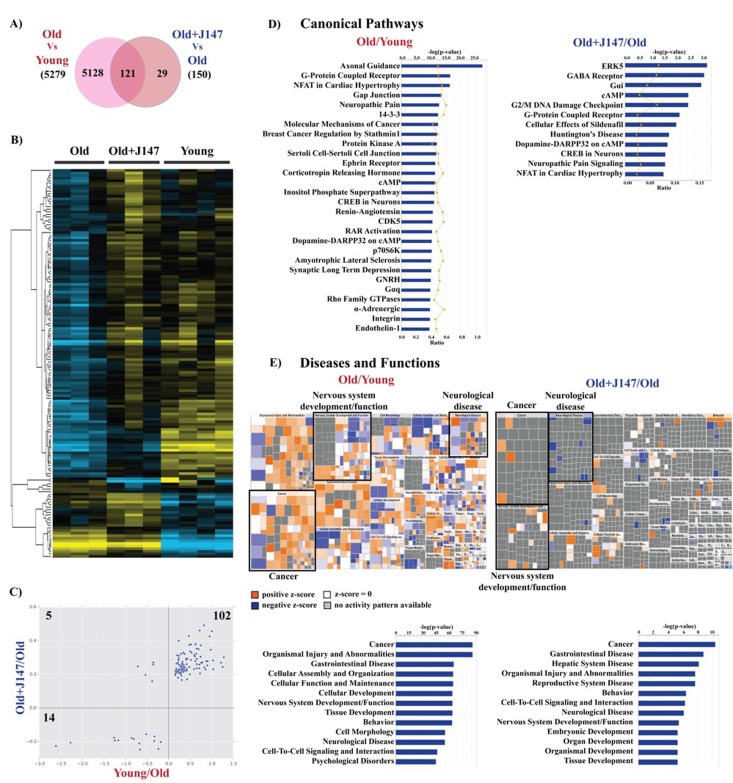
Whole transcriptome analysis of hippocampus shows a rescue of some age-related changes in RNA expression by J147 (**A**) Venn diagram illustrating shared and uniquely affected genes. (**B**) Heatmap of the 150 genes found significantly modified between Old, Old+J147 and Young SAMP8 mice. Scaled expression value (Z-score) is plotted in yellow-blue color scale with yellow indicating high expression and blue indicating low expression. (n = 3-4/group). (**C**) Correlation of gene expression altered in Young/Old and Old+J147/Old (units are -log(fold change)). Predicted canonical biological pathways (**D**) and diseases/functions (**E**) associated with the alterations in gene expression. Only the top significant pathways are indicated.

To elucidate how changes in behavior, proteomics and metabolomics are related to alterations in gene expression, whole transcriptome analysis was carried out with hippocampal tissue. 5279 genes were altered between the old and young SAMP8 mice and 150 genes were changed with J147 treatment (Fig. 7 A). The heatmap in Figure 7 B shows a rescuing effect by J147 of changes verified between the young and old SAMP8 mice. Correlation of the expression of the overlapping 121 genes between the two comparisons confirms that most of these (116 genes; 77% of total genes changed with J147) are indeed associated with a rescue of age-related changes in gene expression (Fig. 7 C).

The IPA of canonical pathways revealed a number of important signaling pathways related to brain function, including axonal guidance, G-protein coupled receptor (GPCR), protein kinase A (PKA), cAMP and neuronal cAMP response element-binding protein (CREB) signaling (Fig. 7 D). Importantly, treatment with J147 altered the expression of genes associated with some of these pathways, namely cAMP, GPCR and CREB signaling. Also of interest are the changes in the renin-angiotensin and endothelin-1 signaling in old SAMP8 mice, which are important regulators of vascular function, supporting the Western blotting data regarding alterations in the brain vasculature (Fig. 4 A and B).

The large dataset obtained with the whole transcriptome allowed for a more informative prediction on the diseases and functions associated with aging. These included cancer as well as neurological disorders and nervous system homeostasis. The mosaics (Fig. 7 E) depict the activation state of specific components in each disease/function group. It is worth noting that the changes associated with J147 are related to decreases in neurological disease and increases in neuronal function and cancer signaling. The whole transcriptome data also correlated well with the data obtained using the Nano-string technology for inflammatory genes (not shown).

In summary, the RNA analysis strongly complements the rest of the data presented here and further supports the potential protective effects of J147 on the central nervous system (CNS) by virtue of its ability to rescue specific aspects of aging that are associated with CNS dysfunction with particular relevance to AD.

## DISCUSSION

We used an integrated multiomics approach to investigate the interaction at the molecular level between an AD drug candidate and aging in the SAMP8 model of aging and early sporadic AD. Our data show that the detrimental changes in behavior that occur with age are accompanied by alterations in protein and RNA expression, as well as in the levels of many key metabolites. Most importantly, our study identifies a large set of parameters in old SAMP8 mice that are associated with stress, vascular pathology, and inflammation that are also observed in human aging and AD. J147 prevented the alteration of many of the metabolic parameters of aging as well as features directly related to the clinical hallmarks of AD, including memory impairment, Aβ content and tau hyperphosphorylation.

SAMP8 mice develop a progressive, age-associated decline in brain function as well as pathophysiological features similar to those found in the brains of sporadic AD patients. Therefore, they may represent an excellent model for studying the relationship between aging and sporadic AD [5, 6]. Our data greatly expand the existing knowledge of the age and AD-associated SAMP8 phenotype at both the brain and system levels. Since the positive effects of J147 in old SAMP8 mice included an improvement in physical and cognitive parameters, reflecting a preservation of their health in general, our results suggest that J147 might be acting by preventing specific metabolic changes that result as a consequence of old age-associated stress.

HSPs represent a major cellular defense against the proteotoxic stress that is characteristic of age-related neurodegenerative disorders. HSP expression depends upon the type of HSP, the disease, cell type and brain region [23]. The changes in HSP40, 60 and 90 observed in the hippocampus of old SAMP8 mice are indicative of stress, and J147 returned the levels of HSP60 and 90 to those of young mice. eIF2α is also involved in protein homeostasis and potentially AD [9]. J147 returned both its level and phosphorylation state to those of young animals.

One of the most prominent manifestations of stress during aging is the production of inflammatory mediators accompanied by metabolic alterations. Although clinical trials with a few anti-inflammatory drugs failed to prevent AD disease progression, epidemiological studies suggest that long-term use of anti-inflammatory drugs may reduce the risk [12]. The data presented here show an increase in inflammatory parameters in old SAMP8 mice. In the CNS, inflammation is often characterized by the activation of glial cells, mainly astrocytes and microglia. This age-associated phenotype is characteristic of the AD brain [24]. SAMP8 mice also develop astrogliosis [7], and we demonstrate here that J147 reduces astrocytic reactivity. Microglia are the resident macrophages of the brain and play a central role during inflammation in the aging and AD brains [25]. Although J147 did not reduce the increased number of microglia cells found in the hippocampus of old SAMP8 mice, it reduced activation of the stress-induced SAPK/JNK as well as the RNA expression of a number of markers of inflammation in the brain. Activation of SAPK/JNK is associated with the inflammatory response in activated microglia [26] and it is possible that J147 preserved microglial function without affecting their number.

Interestingly, the altered expression of several components of the complement system in old SAMP8 mice was not changed by J147. This is important given that the complement system is also activated in human AD patients but that it is thought to be part of a neuroprotective response that helps clear apoptotic cells and Aβ peptide [27]. Therefore, there are parts of the innate immune system that may be beneficial in the context of aging and AD. The fact that J147 did not alter the levels of these components yet reduced Aβ levels, inflammation and vascular pathology supports this idea.

A potentially harmful aspect of inflammation in the aging brain is that it may lead to the impairment of vascular integrity and alterations in neuronal homeostasis [13]. Accordingly, aged SAMP8 mice display disrupted BBB permeability [14]. Our data strongly support the idea that neurovascular dysregulation is an important pathophysiological feature of brains from old SAMP8 mice, and that J147 may protect brain function in these old mice at least in part by preserving BBB homeostasis. The evidence includes the reduction in the levels of VCAM-1, the prevention of IgG infiltration into the hippocampus and the modulation of eicosanoids that regulate vascular dynamics.

Also important is the observation that J147 significantly increased the levels of DHA in the brain. DHA is the primary structural fatty acid in the human brain and has been linked to cognitive performance. While low plasma levels of DHA are associated with cognitive decline in elderly and AD patients, higher DHA intake and plasma levels inversely correlate with AD risk [28]. DHA supplementation in aged animals enhances learning and memory, and protects against Aβ and tau pathology in AD mouse models [28–30]. The increase in DHA with J147 could be a consequence of its reduced oxidation, as suggested by lower levels of its oxidized metabolites HDoHes. Furthermore, a dramatic reduction of other oxidized eicosanoids was also observed with J147 treatment.

Aging is accompanied by strong metabolic alterations at the organismal level that are often associated with mitochondrial dysfunction [21, 31]. Our analysis of the metabolomic profile of the plasma of old SAMP8 mice revealed profound changes in several biological pathways in comparison to the young SAMP mice. The levels of several amino acids and peptides were found lowered in the old SAMP8 mice. These included γ-glutamyl amino acids, dipeptides and the BCAAs. γ-glutamyl amino acids are generated during the γ-glutamyl cycle, which is involved in the transport of glutathione (GSH) between different organs [32]. GSH is the major intracellular antioxidant. Extracellular GSH is usually broken down to its constituent amino acids by the enzyme γ-glutamyl transpeptidase (GGT) and then those are transported across the plasma membrane to regenerate GSH intracellularly [32]. This enzymatic reaction transfers the γ-glutamyl moiety from GSH to acceptor amino acids. The fact that several γ-glutamyl amino acids were lower in the plasma of old SAMP8 could be related to impaired GSH homeostasis in the mice.

Amino acid metabolism is of particular relevance to aging because studies with animals and humans strongly suggest that dietary supplementation with essential and branched chain amino acids positively affects physical health and promotes survival [33]. It was proposed that the positive effects of BCAAs are directly associated with improvement of mitochondrial function [33]. J147 restored to young levels many of the alterations in γ-glutamyl amino acids and BCAAs found in the old SAMP8 mice, as well as the levels of several dipeptides.

In a study that compared the plasma metabolome of young with older mice, there was an increase in fatty acids (C18:0 and C20:4(6) with age [34]. Similarly, old SAMP8 mice have significantly increased levels of both C18:0 and C20:4(6 (Table S1) as well as many other fatty acids, in particular a large group of PUFAs. However, in contrast with the earlier report, we found striking increases in the levels of several acylcarnitines. This is a very important observation because fatty acids must be transported into the mitochondria using the carnitine shuttle in order to produce energy via β-oxidation. When mitochondrial β-oxidation is defective, plasma levels of acylcarnitines rise, as we found in old SAMP8 mice. That J147 restored the levels of acylcarnitines suggests a positive effect on mitochondrial dynamics. In addition, it has been shown that acylcarnitines can directly activate pro-inflammatory pathways [35], supporting the idea that J147 also reduces stress-associated inflammation in old age at the systemic level. These data are strengthened by the IPA, which identified diseases related to hepatic dysfunction and inflammatory responses associated with these metabolic alterations.

The analysis of brain metabolites revealed an alteration in glycolysis and the TCA cycle in old SAMP8 mice, further supporting the idea that mitochondrial function is affected with old age. Our data are in accordance with a recent study comparing the metabolome of plasma and CSF of AD patients and cognitively normal age-matched controls, where disturbances in multiple pathways related to energy metabolism and mitochondrial function were identified [36].

J147 had an impact on some of the metabolites within the brain, but this was not as pronounced as those found in the plasma. However, it did restore the reduced levels of glutamate detected in old mice to that of young animals. Glutamate can be synthesized from the TCA intermediate α-ketoglutarate, which was also restored by J147. Glutamate is the major neurotransmitter in the brain and is involved in learning and memory processes. Studies have shown a decrease in brain glutamate levels with aging [37], as well as in the AD brain [38, 39].

Another metabolite altered in the hippocampus of old SAMP8 mice whose levels were restored by J147 was cAMP. Upregulation of cAMP signaling has been implicated in AD physiopathology and increased cAMP-PKA signaling in the aging brain is associated with impaired cognition and increased vulnerability to neurodegeneration [22]. PKA is a tau kinase and its dysregulation might be partially responsible for AD-related abnormal tau phosphorylation [40], which could explain in part its increased phosphorylation in the hippocampus of old SAMP8 mice and the protection by J147. PKA signaling was also one of the canonical pathways predicted by the RNA IPA to be affected in old mice.

The relevance of cAMP in the old SAMP8 mice and the therapeutic effect of J147 were validated by the transcriptomic analysis, which identified the cAMP and CREB signaling pathways as being significantly altered. This is a good example of how omic approaches that target different cell physiological components can complement each other in order to provide solid readouts about relevant pathways. Importantly, changes given by J147 were associated with both the cAMP and CREB signaling pathways, suggesting that J147 might be exerting its protective effects in the brain by preserving the proper function of these pathways. The latter result is in agreement with our previous data showing that CREB, a transcription factor with a crucial role in neuronal plasticity and memory, is activated by J147 [3].

Cancer was one of the top diseases associated with aging predicted by the IPA from both the RNA and metabolite data. Although we have seen no indication of tumorigenesis in our SAMP8 model, it is known that the incidence of cancer increases with age [41]. The changes reported here may thus reflect metabolic alterations associated with cellular senescence, a known contributor to cancer development [41].

An important observation derived from our multiomics approach is that both the metabolomic and transcriptomic analyses revealed that the vast majority of the changes associated with J147 treatment rescued physiological alterations that were observed with aging. These findings strongly suggest that J147 might be lowering the AD-related pathology in old SAMP8 mice by preventing some of the deterioration associated with aging.

The purpose of this study was to combine data derived from different technological approaches to assess the relationship between aging and the therapeutic effects of J147 in the SAMP8 mouse model of aging and sporadic AD. In this regard, the RNA data showed alterations associated with behavior, dysfunction of the nervous system and neurological disease. These predictions given by the IPA not only are in agreement with those found in the metabolite analysis, but also are consistent with the cognitive impairment assessed by the behavioral testing and the changes in proteins required for synaptic function and relevant to AD assessed by Western blotting. The IPA also identified altered pathways in the old SAMP8 mice linked to vascular homeostasis, which are consistent with the increased vascular inflammation and BBB disruption found with aging, as well as with the changes in eicosanoids involved in vascular function.

Overall, the integration of the data acquired with the multiple scientific techniques applied here not only allowed us to identify with a high level of confidence specific pathologies and molecular pathways characteristic of old age and critical in AD, but also to define a subset that are reverted to youthful levels by J147, suggesting that J147 may be effective at treating the primary causes of the disease. Finally, our study strongly supports the use of this and other rodent models of aging that develop AD-related pathology [42], to address fundamental questions and test new therapeutic interventions.

## METHODS

### Study design

The aim of this project was to investigate whether the AD drug candidate J147 protects SAMP8 mice from aging and AD-associated pathology and to assay the associated metabolic changes. Seventeen three-month old male SAMP8 mice were fed with control diet (LabDiet 5015, TestDiet, Richmond, IN) and eighteen three-month old male SAMP8 mice were fed with J147 diet (LabDiet 5015 + 200ppm J147, TestDiet) until they reached ten months old. At this age, SAMP8 mice present a strong phenotype [7]. The dose of J147 used was 200 ppm (~10mg/kg/day), which previously proved effective in AD transgenic mice [2, 3]. Fourteen three-month old male SAMP8 mice were used as the young control group. The SAMP8 mice are an inbred strain and, as such, young SAMP8 mice were chosen as controls for young age. Given the seven month duration of the feeding paradigm, the effect of J147 diet could only be assessed in old SAMP8 mice, and any age-related changes defined by the comparison to the young SAMP8 animals. All mice were randomly assigned to experimental groups. The number of mice per group was determined based on previous experiments [7] and was sufficient to attain statistical power. Six old SAMP8 mice fed with control diet and two old SAMP8 mice fed with J147 diet died throughout the course of this study. Behavioral testing was carried out one month prior to sacrifice and collection of biological material. Data were analysed by blinded researchers when appropriate.

### SAMP8 mice

The SAMP8 line was acquired from Harlan Laboratories (U.K.). Mouse body weights were measured regularly and no significant differences were found between the groups (Fig. S1). All experiments were performed in accordance with the US Public Health Service Guide for Care and Use of Laboratory Animals and protocols approved by the IACUC at the Salk Institute.

### Behavioral assays

For detailed descriptions of the materials and methods regarding the open field, the elevated plus maze, the object recognition test and the Barnes maze assays, please see supplemental information.

### Tissue preparation

Mice were anesthetized and their blood collected by cardiac puncture. After perfusing with PBS, their brains were removed. Half of the brain was fixed and processed for histology and the other half was dissected (to collect cortex and hippocampus) and prepared for Western blot (WB), RNA extraction, eicosanoid and metabolomic analysis.

### Western blotting

Western blots were carried out as described previously [43]. A list of the antibodies used can be found in the supplemental information. IgG heavy and light chains were detected by blotting the membrane directly with the mouse secondary antibody.

### Immunohistochemistry

Immunohistochemistry was carried out as described previously [43]. Anti-Iba-1 (#019-19741, 1/4000, from Wako) and biotinylated rabbit secondary antibody (#BA1000, 1/400 from Vector Laboratories) were used. Number of microglia per mm^2^ of hippocampus was quantified using the Image J software (NIH). Total counts in 2-4 sections per eight mouse brains of each group were determined in an unbiased fashion.

### Aβ ELISA

Aβ 1-40 and 1-42 levels in hippocampal lysates were analyzed using the Aβ1−40 and Aβ1-42 ELISA kits from Invitrogen (# KMB3481 and # KMB3442, respectively).

### Eicosanoid analysis

Eicosanoids were prepared and analyzed as described previously [43].

### Metabolomic analysis

Metabolomic analyses were conducted at Metabolon as described previously [44]. For statistical analyses and data display, any missing values were assumed to be below the limits of detection and imputed with the compound minimum (minimum value imputation). An estimate of the false discovery rate (Q-value) was calculated to take into account the multiple comparisons that normally occur in metabolomic-based studies, with Q<0.05 used as an indication of high confidence in a result.

### RNA analysis

RNA was isolated from hippocampus using the RNeasy Plus Universal mini kit (Qiagen) and RNA analysis performed by Nanostring.

#### Nanostring

The nCounter GX Mouse Inflammation Kit (Nanostring, Seattle, USA) was used to measure a comprehensive set of 248 inflammation related mouse genes and six internal reference genes.

#### Whole transcriptome analysis

RNA-Seq libraries were prepared using the Illumina TruSeq Stranded mRNA Sample Prep Kit according to the manufacturer's instructions. Briefly, poly-A RNA was selected using poly dT-beads. mRNA was then fragmented and reverse transcribed. cDNA was end-repaired, adenylated and ligated with Illumina adapters with indexes. Adapter-ligated cDNA was then amplified. Libraries were pooled and sequenced single-end 50 base-pair (bp) on the Illumina HiSeq 2500 platform. Sequencing reads were mapped to the mm9 mouse genome using the spliced aligner STAR (2.3.0e) with default parameters [45]. Indices for the alignment were built using the Illumina iGenomes gene annotation for mm9 as a splice junction database and the sjdbOverhang parameter set to 100. Raw gene-level read counts were calculated using this same gene annotation with featureCounts (1.4.6) from the Subread package [46]. Expression normalization and differential analysis were carried out with DESeq2, using a false discovery rate cut-off of 0.1 [47]. The data discussed in this publication have been deposited in NCBI's Gene Expression Omnibus [48] and are accessible through GEO Series accession number GSE69244 (http://www.ncbi.nlm.nih.gov/geo/query/acc.cgi?acc=GSE69244).

### Bioinformatics and statistics

Data in figures is presented as group mean ± SD or as box-and-whisker plots indicating the group minimum, lower quartile, median, upper quartile, and group maximum. Data from the Western blotting and eicosanoids analyses were normalized to the average of the young SAMP8 control group. For metabolites, the measured values across the three groups were median-normalized to 1.

Data were analysed and the functional/network analyses were generated through the use of QIAGEN's Ingenuity Pathway Analysis (IPA^®^, QIAGEN Redwood City, http://www.qiagen.com/ingenuity).

Metaboanalyst [49] was used to generate the heatmaps. Values were mean-centered and divided by the SD of each variable (scaled Z-score). Hierarchical clustering of RNA expression was performed using Euclidean distances and the Ward algorithm.

Statistical analysis of the three groups was carried out by one-way ANOVA followed by Tukey-Kramer multiple comparison *post hoc* test was used. For data regarding multiple time points, two-way repeated-measures ANOVA and post hoc Bonferroni corrected t tests were applied. GraphPad Prism 6 was used and exact *P* values are indicated (for *P* < 0.050). All data are mean ± SD.

## SUPPLEMENTAL INFORMATION TABLES AND FIGURES







## References

[1] Prior M, Chiruta C, Currais A, Goldberg J, Ramsey J, Dargusch R, Maher PA, Schubert D (2014). Back to the future with phenotypic screening. ACS Chem Neurosci.

[2] Chen Q, Prior M, Dargusch R, Roberts A, Riek R, Eichmann C, Chiruta C, Akaishi T, Abe K, Maher P, Schubert D (2011). A novel neurotrophic drug for cognitive enhancement and Alzheimer's disease. PLoS One.

[3] Prior M, Dargusch R, Ehren JL, Chiruta C, Schubert D (2013). The neurotrophic compound J147 reverses cognitive impairment in aged Alzheimer's disease mice. Alzheimers Res Ther.

[4] Swerdlow RH (2007). Is aging part of Alzheimer's disease, or is Alzheimer's disease part of aging?. Neurobiol Aging.

[5] Cheng XR, Zhou WX, Zhang YX (2014). The behavioral, pathological and therapeutic features of the senescence-accelerated mouse prone 8 strain as an Alzheimer's disease animal model. Ageing Res Rev.

[6] Morley JE, Armbrecht HJ, Farr SA, Kumar VB (2012). The senescence accelerated mouse (SAMP8) as a model for oxidative stress and Alzheimer's disease. Biochim Biophys Acta.

[7] Currais A, Prior M, Lo D, Jolivalt C, Schubert D, Maher P (2012). Diabetes exacerbates amyloid and neurovascular pathology in aging-accelerated mice. Aging Cell.

[8] Morley JE, Farr SA, Kumar VB, Armbrecht HJ (2012). The SAMP8 mouse: a model to develop therapeutic interventions for Alzheimer's disease. Curr Pharm Des.

[9] Chang RC, Wong AK, Ng HK, Hugon J (2002). Phosphorylation of eukaryotic initiation factor-2alpha (eIF2alpha) is associated with neuronal degeneration in Alzheimer's disease. Neuroreport.

[10] Thinakaran G, Koo EH (2008). Amyloid precursor protein trafficking, processing, and function. J Biol Chem.

[11] Ikura Y, Kudo T, Tanaka T, Tanii H, Grundke-Iqbal I, Iqbal K, Takeda M (1998). Levels of tau phosphorylation at different sites in Alzheimer disease brain. Neuroreport.

[12] Wyss-Coray T, Rogers J (2012). Inflammation in Alzheimer disease-a brief review of the basic science and clinical literature. Cold Spring Harb Perspect Med.

[13] Grammas P (2011). Neurovascular dysfunction, inflammation and endothelial activation: implications for the pathogenesis of Alzheimer's disease. J Neuroinflammation.

[14] Pelegri C, Canudas AM, del Valle J, Casadesus G, Smith MA, Camins A, Pallas M, Vilaplana J (2007). Increased permeability of blood-brain barrier on the hippocampus of a murine model of senescence. Mech Ageing Dev.

[15] Rodriguez JJ, Olabarria M, Chvatal A, Verkhratsky A (2009). Astroglia in dementia and Alzheimer's disease. Cell Death Differ.

[16] Ploia C, Antoniou X, Sclip A, Grande V, Cardinetti D, Colombo A, Canu N, Benussi L, Ghidoni R, Forloni G, Borsello T (2011). JNK plays a key role in tau hyperphosphorylation in Alzheimer's disease models. J Alzheimers Dis.

[17] Buczynski MW, Dumlao DS, Dennis EA (2009). Thematic Review Series: Proteomics. An integrated omics analysis of eicosanoid biology. J Lipid Res.

[18] Morin C, Fortin S, Rousseau E (2011). 19,20-EpDPE, a bioactive CYP450 metabolite of DHA monoacyglyceride, decreases Ca(2)(+) sensitivity in human pulmonary arteries. Am J Physiol Heart Circ Physiol.

[19] Viswanathan S, Hammock BD, Newman JW, Meerarani P, Toborek M, Hennig B (2003). Involvement of CYP 2C9 in mediating the proinflammatory effects of linoleic acid in vascular endothelial cells. J Am Coll Nutr.

[20] Hardwick JP, Eckman K, Lee YK, Abdelmegeed MA, Esterle A, Chilian WM, Chiang JY, Song BJ (2013). Eicosanoids in metabolic syndrome. Adv Pharmacol.

[21] Lin MT, Beal MF (2006). Mitochondrial dysfunction and oxidative stress in neurodegenerative diseases. Nature.

[22] Carlyle BC, Nairn AC, Wang M, Yang Y, Jin LE, Simen AA, Ramos BP, Bordner KA, Craft GE, Davies P, Pletikos M, Sestan N, Arnsten AF (2014). cAMP-PKA phosphorylation of tau confers risk for degeneration in aging association cortex. Proc Natl Acad Sci U S A.

[23] Leak RK (2014). Heat shock proteins in neurodegenerative disorders and aging. J Cell Commun Signal.

[24] Li C, Zhao R, Gao K, Wei Z, Yin MY, Lau LT, Chui D, Hoi Yu AC (2011). Astrocytes: implications for neuroinflammatory pathogenesis of Alzheimer's disease. Curr Alzheimer Res.

[25] Mosher KI, Wyss-Coray T (2014). Microglial dysfunction in brain aging and Alzheimer's disease. Biochem Pharmacol.

[26] Waetzig V, Czeloth K, Hidding U, Mielke K, Kanzow M, Brecht S, Goetz M, Lucius R, Herdegen T, Hanisch UK (2005). c-Jun N-terminal kinases (JNKs) mediate pro-inflammatory actions of microglia. Glia.

[27] Rubio-Perez JM, Morillas-Ruiz JM (2012). A review: inflammatory process in Alzheimer's disease, role of cytokines. ScientificWorldJournal.

[28] Yurko-Mauro K (2010). Cognitive and cardiovascular benefits of docosahexaenoic acid in aging and cognitive decline. Curr Alzheimer Res.

[29] Green KN, Martinez-Coria H, Khashwji H, Hall EB, Yurko-Mauro KA, Ellis L, LaFerla FM (2007). Dietary docosahexaenoic acid and docosapentaenoic acid ameliorate amyloid-beta and tau pathology via a mechanism involving presenilin 1 levels. J Neurosci.

[30] Lim GP, Calon F, Morihara T, Yang F, Teter B, Ubeda O, Salem N, Frautschy SA, Cole GM (2005). A diet enriched with the omega-3 fatty acid docosahexaenoic acid reduces amyloid burden in an aged Alzheimer mouse model. J Neurosci.

[31] Navarro A, Boveris A (2010). Brain mitochondrial dysfunction in aging, neurodegeneration, and Parkinson's disease. Front Aging Neurosci.

[32] Zhang H, Forman HJ, Choi J (2005). Gamma-glutamyl transpeptidase in glutathione biosynthesis. Methods Enzymol.

[33] Valerio A, D'Antona G, Nisoli E (2011). Branched-chain amino acids, mitochondrial biogenesis, and healthspan: an evolutionary perspective. Aging (Albany NY).

[34] Houtkooper RH, Argmann C, Houten SM, Canto C, Jeninga EH, Andreux PA, Thomas C, Doenlen R, Schoonjans K, Auwerx J (2011). The metabolic footprint of aging in mice. Sci Rep.

[35] Rutkowsky JM, Knotts TA, Ono-Moore KD, McCoin CS, Huang S, Schneider D, Singh S, Adams SH, Hwang DH (2014). Acylcarnitines activate proinflammatory signaling pathways. Am J Physiol Endocrinol Metab.

[36] Trushina E, Dutta T, Persson XM, Mielke MM, Petersen RC (2013). Identification of altered metabolic pathways in plasma and CSF in mild cognitive impairment and Alzheimer's disease using metabolomics. PLoS One.

[37] Zahr NM, Mayer D, Pfefferbaum A, Sullivan EV (2008). Low striatal glutamate levels underlie cognitive decline in the elderly: evidence from in vivo molecular spectroscopy. Cereb Cortex.

[38] Fayed N, Modrego PJ, Rojas-Salinas G, Aguilar K (2011). Brain glutamate levels are decreased in Alzheimer's disease: a magnetic resonance spectroscopy study. Am J Alzheimers Dis Other Demen.

[39] Rupsingh R, Borrie M, Smith M, Wells JL, Bartha R (2011). Reduced hippocampal glutamate in Alzheimer disease. Neurobiol Aging.

[40] Liu F, Liang Z, Shi J, Yin D, El-Akkad E, Grundke-Iqbal I, Iqbal K, Gong CX (2006). PKA modulates GSK-3beta- and cdk5-catalyzed phosphorylation of tau in site- and kinase-specific manners. FEBS Lett.

[41] de Magalhaes JP (2013). How ageing processes influence cancer. Nat Rev Cancer.

[42] Stefanova NA, Muraleva NA, Korbolina EE, Kiseleva E, Maksimova KY, Kolosova NG (2015). Amyloid accumulation is a late event in sporadic Alzheimer's disease-like pathology in nontransgenic rats. Oncotarget.

[43] Currais A, Prior M, Dargusch R, Armando A, Ehren J, Schubert D, Quehenberger O, Maher P (2014). Modulation of p25 and inflammatory pathways by fisetin maintains cognitive function in Alzheimer's disease transgenic mice. Aging Cell.

[44] Shin SY, Fauman EB, Petersen AK, Krumsiek J, Santos R, Huang J, Arnold M, Erte I, Forgetta V, Yang TP, Walter K, Menni C, Chen L (2014). An atlas of genetic influences on human blood metabolites. Nat Genet.

[45] Dobin A, Davis CA, Schlesinger F, Drenkow J, Zaleski C, Jha S, Batut P, Chaisson M, Gingeras TR (2013). STAR: ultrafast universal RNA-seq aligner. Bioinformatics.

[46] Liao Y, Smyth GK, Shi W (2014). featureCounts: an efficient general purpose program for assigning sequence reads to genomic features. Bioinformatics.

[47] Love MI, Huber W, Anders S (2014). Moderated estimation of fold change and dispersion for RNA-seq data with DESeq2. Genome Biol.

[48] Edgar R, Domrachev M, Lash AE (2002). Gene Expression Omnibus: NCBI gene expression and hybridization array data repository. Nucleic Acids Res.

[49] Xia J, Mandal R, Sinelnikov IV, Broadhurst D, Wishart DS (2012). MetaboAnalyst 2.0--a comprehensive server for metabolomic data analysis. Nucleic Acids Res.

