# Supplementary material for: A comprehensive multiomics approach toward understanding the relationship between aging and dementia
Source: Aging (Albany NY). 2015 Nov 11;7(11):937–52. doi: 10.18632/aging.100838 (PMC4694064; doi:10.18632/aging.100838)
Supplement: Supplementary file 1 [file aging-07-0937-s001.pdf]

SUPPLEMENTAL INFORMATION

SUPPLEMENTAL METHODS

Open field. The open field test was performed using MED Associates hardware and the Activity Monitor software according to the manufacturer's instructions (MED Associates Inc, St. Albans, VT, USA). Animals were individually placed into clear Plexiglas boxes (40.6 x 40.6 x 38.1 cm) surrounded by multiple bands of photo beams and optical sensors that measure horizontal and vertical activity. Their movement was detected as breaks within the beam matrices and automatically recorded for 30 minutes.

Elevated plus maze. The maze consisted of four arms (two open without walls and two enclosed by 15.25 cm high walls) 30 cm long and 5 cm wide in the shape of a plus. A video-tracking system (Noldus EthoVision) was used to automatically collect behavioral data. The software was installed on a PC computer with a digital video camera mounted overhead on the ceiling, which automatically detected and recorded when mice entered the open or closed arms of the maze and the time spent in each. Mice were habituated to the room 24 hr before testing and habituated to the maze for 1 min before testing by placing them in the center of the maze and blocking entry to the arms. Mice were then tested for a 5 min period and their behaviour recorded. Disinhibition was measured by comparing the time spent on the open arms to time spent on the closed arms.

Object recognition. Mice were tested in a standard home cage. Phase 1 (Habituation): Each mouse was placed into the apparatus (no objects present) for two 10 min sessions separated by 1-4 hours to habituate to testing environment. Phase 2 (Training): Two identical Velcro-backed objects (object "A") were attached into designated corners of the apparatus. The mouse was placed into the apparatus opposite to the objects and recorded by a camera for 10 min. Phase 3 (Test): One hour after training, the test phase began. Only one of the objects was replaced with a new object (object "B"). The mouse was placed into the apparatus opposite to the objects and recorded for 5 min. The apparatus was wiped and objects cleaned with 70% alcohol to remove odors between mice. "object recognition index" was calculated by dividing the amount of time spent with object B by the total time spent with objects A + B and multiplied by 100.

Barnes maze. The maze consisted of a flat circular surface (36" diameter) with 20 equally spaced holes (2" diameter) along the outer edge. One of the holes led to a dark hide box while the other 19 led to false boxes that were too small to be entered. The latency to enter the hide box was recorded. The test was conducted in three phases. Phase 1 (Training): A hide box was placed under one of the holes. Animals were placed into an opaque cylinder in the center of the maze for 30 sec to promote spatial disorientation at the start of the test. After 30 sec, the cylinder was removed and the animal explored the maze until it found and entered the hide box. The number of incorrect entries was scored. If the mouse failed to enter the box within 3 min, it was gently led into the box. The animal remained in the box for an additional 20 sec before it was removed from the boxed and gently placed into the home cage. Training is repeated three times a day for four days. The location of the hide box remained the same during every trial but it was shifted between subjects to reduce the potential for unintended intra-maze cues. Phase 2 (Retention): This phase measures retention of spatial memory following a delay. After a two day break from training, each animal was re-tested for a one day, three-trial session using the same hide box location as before. Phase 3 (Reversal): This phase examines memory reversal. On the day following the retention phase, a new hide box location was established 180 degrees to the original location. The same method as before was used and trials were repeated three times a day over two consecutive days.

Western blotting. The primary antibodies used were: 5-LOX (#610694, 1/1000) from BD Biosciences; eIF2α (#2103, 1/5000), phospho-eIF2α Ser51 (#9721, 1/1000), HRP-conjugated rabbit anti-actin (#5125, 1/20,000), HSP40 (#4871, 1/1000), HSP60 (#4870, 1/5000), HSP70 (#4872, 1/5000), HSP90 (#4877, 1/5000), SAPK/JNK (#9252, 1/1000), Phospho-SAPK/JNK Thr183/Tyr185 (#4668, 1/1000), phospho-tau Ser396 (#9632, 1/1000), from Cell Signaling Technology; tau (#A0024, 1/10000), from Dako Cytomation; Arc (#sc-15325, 1/2000), GFAP (#AB5804, 1/5000), SAP102 (#AB5170, 1/400), from Millipore; VCAM-1 (#sc-1504, 1/500) from Santa Cruz Biotechnology; APP C-Terminal (#A8717, 1/100000), from Sigma. Horseradish peroxidase-conjugated secondaries goat anti-rabbit, goat anti-mouse or rabbit anti-goat (Biorad) diluted 1/5000 were used.

SUPPLEMENTAL TABLES

Please browse the links in full text version of this manuscript to see the Supplemental Tables:

Table S1: List of 195 (out of 593) biochemicals found significantly altered in the blood plasma of young SAMP8, old SAMP8 and old SAMP8 mice fed J147. Fold changes and specific *P* values are indicated. One-way ANOVA followed by Tukey-Kramer post-hoc test (n = 5/group).

Table S2: List of 105 (out of 493) biochemicals found significantly altered in the brain cortex of young SAMP8, old SAMP8 and old SAMP8 mice fed J147. Fold changes and specific *P* values are indicated. One-way ANOVA followed by Tukey-Kramer post-hoc test (n = 5/group).

SUPPLEMENTAL FIGURES

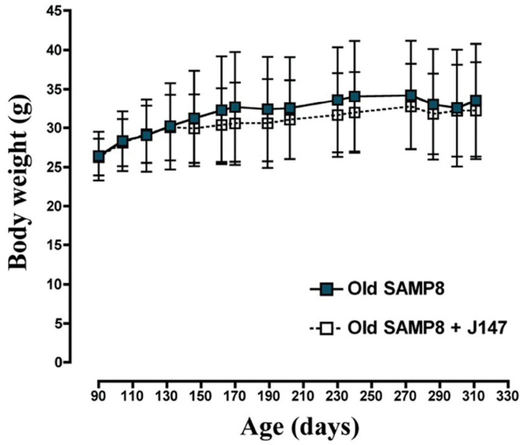

Figure S1. Body weights of SAMP8 mice fed with vehicle and J147 diets. Three-month old SAMP8 mice were fed with vehicle (n = 17) or J147 (n = 18) diets until ten months old. Six SAMP8 mice fed with vehicle diet and two SAMP8 mice fed with J147 diet died during the course of this study. No significant differences were found between the body weights of the two groups. Two-way repeated measures ANOVA and post hoc Bonferroni corrected t-test. All data are mean ± SD.

Plasma

A) Number Biochemicals/Group Altered

Old Vs Young

>	Leucine, Isoleucine and Valine Metabolism	14/30
	Lysolipid	12/71
>	Dipeptide	10/16
>	Gamma-glutamyl Amino Acid	9/14
>	Polyunsaturated Fatty Acid (n3 and n6)	9/14
	Phenylalanine and Tyrosine Metabolism	8/24
	Lysine Metabolism	7/11
	Tryptophan Metabolism	7/16
	Food Component/Plant	7/28
	Urea cycle; Arginine and Proline Metabolism	6/12
	Fatty Acid, Dicarboxylate	6/15
	Benzoate Metabolism	6/17
>	Fatty Acid Metabolism (Acyl Carnitine)	5/14
	Methionine, Cysteine, SAM and Taurine Metabolism	5/16
	Chemical	5/19
	TCA Cycle	4/8
	Drug	3/4
	Alanine and Aspartate Metabolism	3/6
	Glycine, Serine and Threonine Metabolism	3/10
	Pentose Metabolism	3/10
	Long Chain Fatty Acid	3/15
	Riboflavin Metabolism	2/2
	Fatty Acid Metabolism (Acyl Glycine)	2/4
	Inositol Metabolism	2/4
	Pyrimidine Metabolism, Uracil containing	2/4
	Ascorbate and Aldarate Metabolism	2/4
	Purine Metabolism, Guanine containing	2/6
	Nicotinate and Nicotinamide Metabolism	2/7

Old+J147 Vs Old

>	Fatty Acid Metabolism (Acyl Carnitine)	4/14
>	Polyunsaturated Fatty Acid (n3 and n6)	4/14
>	Dipeptide	4/16
	Endocannabinoid	2/6
	Purine Metabolism, Guanine containing	2/6
	Pentose Metabolism	2/10
>	Gamma-glutamyl Amino Acid	2/14
	Fatty Acid, Dicarboxylate	2/15
	Methionine, Cysteine, SAM and Taurine Metabolism	2/16
	Chemical	2/19
>	Leucine, Isoleucine and Valine Metabolism	2/30

B) Canonical Pathways

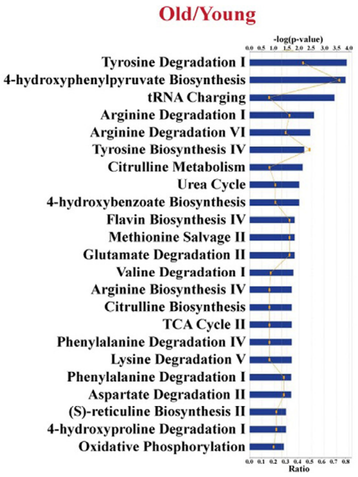

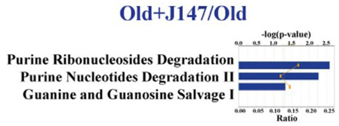

C) Diseases and Functions

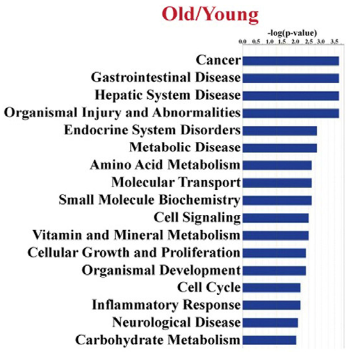

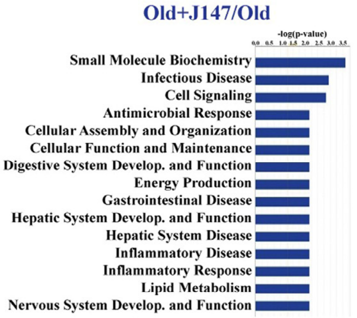

Cortex

D) Number Biochemicals/Group Altered

Old Vs Young

>	Glutamate Metabolism	5/9
>	Glycolysis, Gluconeogenesis, and Pyruvate Metabolism	5/12
	Polyunsaturated Fatty Acid (n3 and n6)	5/13
	Methionine, Cysteine, SAM and Taurine Metabolism	5/16
>	TCA Cycle	4/7
	Food Component/Plant	4/9
	Glycine, Serine and Threonine Metabolism	4/10
>	Urea cycle; Arginine and Proline Metabolism	4/11
	Pantothenate and CoA Metabolism	3/4
	Nucleotide Sugar	3/6
	Fatty Acid, Monohydroxy	3/6
	Phospholipid Metabolism	3/9
	Gamma-glutamyl Amino Acid	3/11
	Leucine, Isoleucine and Valine Metabolism	3/20
	Fatty Acid, Dicarboxylate	2/3
	Aminosugar Metabolism	2/6
	Pentose Metabolism	2/6
	Pentose Phosphate Pathway	2/6
	Lysine Metabolism	2/9
	Phenylalanine and Tyrosine Metabolism	2/10

Old+J147 Vs Old

	Food Component/Plant	3/9
	Purine Metabolism, Adenine containing	3/14
	Leucine, Isoleucine and Valine Metabolism	3/20
>	TCA Cycle	2/7
>	Urea cycle; Arginine and Proline Metabolism	2/11

E) Canonical Pathways

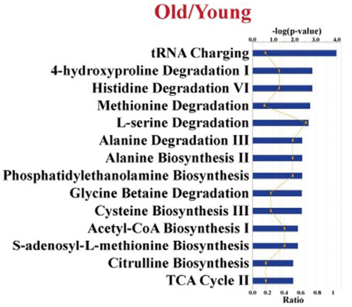

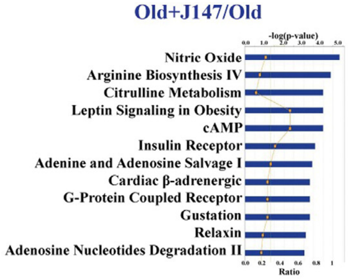

F) Diseases and Functions

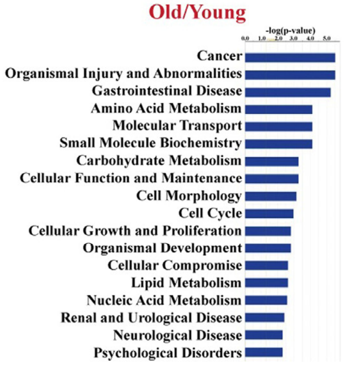

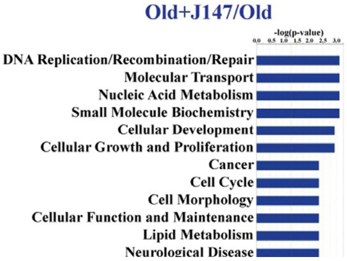

Figure S2. Functional analysis of metabolites found significantly altered. Biochemicals altered in the plasma (**A**) and cortex (**D**) between young and old SAMP8 mice and between old and old SAMP8 mice treated with J147 were organized by descending order according to the number of changes per biological group. In order to provide insight onto the possible biological pathways and diseases/functions associated with the metabolic alterations, Ingenuity Pathway Analysis (IPA) was carried out with the plasma (**B** and **C**) and cortex (**E** and **F**) metabolites present in the HMDB. Only the top significant pathways are indicated.
