# Supplementary material for: A comprehensive multiomics approach toward understanding the relationship between aging and dementia
Source: Aging (Albany NY). 2015 Nov 11;7(11):937–52. doi: 10.18632/aging.100838 (PMC4694064; doi:10.18632/aging.100838)
Supplement: Supplementary file 2 [file aging-07-0937-s002.docx]

| **Supplemental Table 1. Altered biochemicals in the plasma** | | | | | | | | |
| --- | --- | --- | --- | --- | --- | --- | --- | --- |
|  | | | **Old/Young** | | **J147/Old** | | **J147/Young** | |
| **BIOCHEMICAL** | **SUPER PATHWAY** | **SUB PATHWAY** | Fold change | p value | Fold change | p value | Fold change | p value |
| asparagine | Amino Acid | Alanine and Aspartate Metabolism | 0.661 | < 0.001 | 1.213 |  | 0.802 | 0.019 |
| N-acetylalanine | Amino Acid | Alanine and Aspartate Metabolism | 0.713 | 0.005 | 0.926 |  | 0.660 | 0.002 |
| N-acetylasparagine | Amino Acid | Alanine and Aspartate Metabolism | 0.482 | 0.025 | 1.524 |  | 0.734 |  |
| creatinine | Amino Acid | Creatine Metabolism | 0.681 | 0.039 | 1.024 |  | 0.698 |  |
| N-acetylthreonine | Amino Acid | Glycine, Serine and Threonine Metabolism | 0.561 | 0.015 | 0.731 |  | 0.410 | 0.002 |
| dimethylglycine | Amino Acid | Glycine, Serine and Threonine Metabolism | 0.674 | 0.020 | 1.233 |  | 0.832 |  |
| threonine | Amino Acid | Glycine, Serine and Threonine Metabolism | 0.728 | 0.023 | 1.186 |  | 0.863 |  |
| glycine | Amino Acid | Glycine, Serine and Threonine Metabolism | 0.600 |  | 0.723 |  | 0.434 | 0.013 |
| 4-guanidinobutanoate | Amino Acid | Guanidino and Acetamido Metabolism | 0.401 | 0.027 | 1.620 |  | 0.649 |  |
| 1-methylimidazoleacetate | Amino Acid | Histidine Metabolism | 2.376 | 0.017 | 0.703 |  | 1.671 |  |
| 4-imidazoleacetate | Amino Acid | Histidine Metabolism | 1.396 |  | 1.728 |  | 2.413 | 0.018 |
| N-acetyl-3-methylhistidine | Amino Acid | Histidine Metabolism | 1.887 |  | 0.354 | 0.043 | 0.668 |  |
| N-acetylvaline | Amino Acid | Leucine, Isoleucine and Valine Metabolism | 0.509 | < 0.001 | 1.008 |  | 0.513 | < 0.001 |
| N-acetylleucine | Amino Acid | Leucine, Isoleucine and Valine Metabolism | 0.370 | < 0.001 | 1.239 |  | 0.459 | 0.001 |
| beta-hydroxyisovalerate | Amino Acid | Leucine, Isoleucine and Valine Metabolism | 0.443 | < 0.001 | 1.187 |  | 0.526 | 0.001 |
| 3-hydroxyisobutyrate | Amino Acid | Leucine, Isoleucine and Valine Metabolism | 0.483 | 0.001 | 1.387 |  | 0.670 | 0.015 |
| 4-methyl-2-oxopentanoate | Amino Acid | Leucine, Isoleucine and Valine Metabolism | 0.579 | 0.001 | 1.041 |  | 0.603 | 0.002 |
| tiglyl carnitine | Amino Acid | Leucine, Isoleucine and Valine Metabolism | 0.490 | 0.002 | 1.544 |  | 0.756 |  |
| isovalerylcarnitine | Amino Acid | Leucine, Isoleucine and Valine Metabolism | 0.570 | 0.010 | 1.305 |  | 0.743 |  |
| ethylmalonate | Amino Acid | Leucine, Isoleucine and Valine Metabolism | 1.895 | 0.022 | 0.594 | 0.048 | 1.127 |  |
| isovalerylglycine | Amino Acid | Leucine, Isoleucine and Valine Metabolism | 0.543 | 0.040 | 1.388 |  | 0.753 |  |
| isoleucine | Amino Acid | Leucine, Isoleucine and Valine Metabolism | 0.594 | < 0.001 | 1.209 |  | 0.718 | < 0.001 |
| leucine | Amino Acid | Leucine, Isoleucine and Valine Metabolism | 0.594 | < 0.001 | 1.156 |  | 0.686 | < 0.001 |
| N-acetylisoleucine | Amino Acid | Leucine, Isoleucine and Valine Metabolism | 0.373 | < 0.001 | 1.350 |  | 0.504 | 0.001 |
| valine | Amino Acid | Leucine, Isoleucine and Valine Metabolism | 0.616 | < 0.001 | 1.288 | 0.016 | 0.793 | 0.006 |
| tigloylglycine | Amino Acid | Leucine, Isoleucine and Valine Metabolism | 0.329 | < 0.001 | 0.895 |  | 0.294 | < 0.001 |
| alpha-hydroxyisocaproate | Amino Acid | Leucine, Isoleucine and Valine Metabolism | 0.693 |  | 0.781 |  | 0.541 | 0.035 |
| 3-methyl-2-oxovalerate | Amino Acid | Leucine, Isoleucine and Valine Metabolism | 0.733 |  | 0.927 |  | 0.679 | 0.045 |
| N6-acetyllysine | Amino Acid | Lysine Metabolism | 0.689 | 0.001 | 0.991 |  | 0.683 | 0.001 |
| N2,N6-diacetyllysine | Amino Acid | Lysine Metabolism | 0.625 | 0.002 | 1.252 |  | 0.782 |  |
| 2-aminoadipate | Amino Acid | Lysine Metabolism | 0.522 | 0.003 | 1.066 |  | 0.556 | 0.006 |
| N2-acetyllysine | Amino Acid | Lysine Metabolism | 0.634 | 0.004 | 1.015 |  | 0.643 | 0.005 |
| glutarate (pentanedioate) | Amino Acid | Lysine Metabolism | 0.276 | 0.013 | 1.404 |  | 0.388 | 0.024 |
| 2-oxoadipate | Amino Acid | Lysine Metabolism | 0.675 | 0.034 | 0.970 |  | 0.655 | 0.025 |
| pipecolate | Amino Acid | Lysine Metabolism | 0.598 | < 0.001 | 0.815 |  | 0.487 | < 0.001 |
| 2-hydroxybutyrate (AHB) | Amino Acid | Methionine, Cysteine, SAM and Taurine Metabolism | 0.494 | < 0.001 | 1.285 |  | 0.635 | 0.004 |
| N-acetylmethionine sulfoxide | Amino Acid | Methionine, Cysteine, SAM and Taurine Metabolism | 0.413 | 0.003 | 1.417 |  | 0.585 | 0.030 |
| 2-aminobutyrate | Amino Acid | Methionine, Cysteine, SAM and Taurine Metabolism | 0.641 | 0.004 | 1.362 | 0.049 | 0.874 |  |
| N-acetylmethionine | Amino Acid | Methionine, Cysteine, SAM and Taurine Metabolism | 0.532 | 0.013 | 1.284 |  | 0.683 |  |
| methionine | Amino Acid | Methionine, Cysteine, SAM and Taurine Metabolism | 1.640 | 0.015 | 1.045 |  | 1.714 | 0.007 |
| S-methylcysteine | Amino Acid | Methionine, Cysteine, SAM and Taurine Metabolism | 1.433 |  | 1.353 |  | 1.939 | 0.004 |
| N-acetyltaurine | Amino Acid | Methionine, Cysteine, SAM and Taurine Metabolism | 0.773 |  | 0.786 |  | 0.607 | 0.013 |
| N-formylmethionine | Amino Acid | Methionine, Cysteine, SAM and Taurine Metabolism | 1.492 |  | 1.189 | 0.037 | 1.775 |  |
| phenylpyruvate | Amino Acid | Phenylalanine and Tyrosine Metabolism | 0.555 | 0.001 | 1.090 |  | 0.606 | 0.003 |
| 4-hydroxycinnamate | Amino Acid | Phenylalanine and Tyrosine Metabolism | 0.248 | 0.004 | 3.555 | 0.013 | 0.881 |  |
| phenylalanine | Amino Acid | Phenylalanine and Tyrosine Metabolism | 0.808 | 0.007 | 1.068 |  | 0.863 | 0.047 |
| 4-hydroxyphenylpyruvate | Amino Acid | Phenylalanine and Tyrosine Metabolism | 0.257 | 0.014 | 0.949 |  | 0.244 | 0.013 |
| tyrosine | Amino Acid | Phenylalanine and Tyrosine Metabolism | 0.666 | 0.016 | 1.134 |  | 0.755 |  |
| 4-hydroxyphenylacetate | Amino Acid | Phenylalanine and Tyrosine Metabolism | 0.293 | 0.017 | 1.875 |  | 0.549 |  |
| 4-hydroxyphenylacetyl glycine | Amino Acid | Phenylalanine and Tyrosine Metabolism | 0.426 | 0.020 | 1.399 |  | 0.596 |  |
| N-acetyltyrosine | Amino Acid | Phenylalanine and Tyrosine Metabolism | 0.523 | 0.034 | 0.845 |  | 0.442 | 0.014 |
| N-acetylphenylalanine | Amino Acid | Phenylalanine and Tyrosine Metabolism | 0.877 |  | 0.709 |  | 0.622 | 0.031 |
| 3-(4-hydroxyphenyl)lactate | Amino Acid | Phenylalanine and Tyrosine Metabolism | 0.659 |  | 0.818 |  | 0.539 | 0.039 |
| indole-3-carboxylic acid | Amino Acid | Tryptophan Metabolism | 0.357 | 0.001 | 0.756 |  | 0.270 | < 0.001 |
| kynurenate | Amino Acid | Tryptophan Metabolism | 0.343 | 0.003 | 1.089 |  | 0.374 | 0.004 |
| xanthurenate | Amino Acid | Tryptophan Metabolism | 0.294 | 0.005 | 1.982 |  | 0.582 |  |
| picolinate | Amino Acid | Tryptophan Metabolism | 0.583 | 0.008 | 1.486 |  | 0.867 |  |
| N-acetyltryptophan | Amino Acid | Tryptophan Metabolism | 0.682 | 0.026 | 0.912 |  | 0.622 | 0.009 |
| anthranilate | Amino Acid | Tryptophan Metabolism | 0.646 | 0.027 | 1.019 |  | 0.658 | 0.032 |
| indoleacetate | Amino Acid | Tryptophan Metabolism | 0.528 | 0.030 | 1.571 |  | 0.830 |  |
| N-acetylkynurenine (2) | Amino Acid | Tryptophan Metabolism | 0.868 |  | 0.833 |  | 0.722 | 0.038 |
| homocitrulline | Amino Acid | Urea cycle; Arginine and Proline Metabolism | 0.427 | 0.001 | 1.218 |  | 0.520 | 0.004 |
| urea | Amino Acid | Urea cycle; Arginine and Proline Metabolism | 0.682 | 0.002 | 1.145 |  | 0.781 | 0.022 |
| ornithine | Amino Acid | Urea cycle; Arginine and Proline Metabolism | 0.666 | 0.002 | 1.070 |  | 0.712 | 0.006 |
| N-acetylarginine | Amino Acid | Urea cycle; Arginine and Proline Metabolism | 0.498 | 0.004 | 1.625 | 0.049 | 0.809 |  |
| pro-hydroxy-pro | Amino Acid | Urea cycle; Arginine and Proline Metabolism | 0.440 | 0.004 | 0.929 |  | 0.409 | 0.003 |
| trans-4-hydroxyproline | Amino Acid | Urea cycle; Arginine and Proline Metabolism | 0.349 | < 0.001 | 1.248 |  | 0.435 | < 0.001 |
| cyclo(L-phe-L-pro) | Peptide | Dipeptide | 0.366 | 0.001 | 1.898 | 0.045 | 0.694 |  |
| glycylvaline | Peptide | Dipeptide | 0.415 | 0.001 | 1.127 |  | 0.468 | 0.002 |
| cyclo(gly-pro) | Peptide | Dipeptide | 0.316 | 0.001 | 2.244 | 0.048 | 0.708 |  |
| pyroglutamylvaline | Peptide | Dipeptide | 0.195 | 0.002 | 2.076 |  | 0.406 | 0.016 |
| isoleucylglycine | Peptide | Dipeptide | 0.401 | 0.003 | 1.163 |  | 0.466 | 0.007 |
| glycylproline | Peptide | Dipeptide | 0.276 | 0.005 | 1.878 |  | 0.519 |  |
| cyclo(leu-pro) | Peptide | Dipeptide | 0.408 | 0.009 | 2.284 | 0.019 | 0.932 |  |
| isoleucylaspartate | Peptide | Dipeptide | 0.422 | 0.012 | 1.553 |  | 0.655 |  |
| cis-Cyclo[L-ala-L-Pro] | Peptide | Dipeptide | 0.501 | 0.013 | 2.099 | 0.007 | 1.052 |  |
| glycylleucine | Peptide | Dipeptide | 0.442 | 0.032 | 1.318 |  | 0.583 |  |
| carnosine | Peptide | Dipeptide Derivative | 0.389 | 0.006 | 1.251 |  | 0.486 | 0.018 |
| gamma-glutamylphenylalanine | Peptide | Gamma-glutamyl Amino Acid | 0.481 | 0.004 | 1.813 | 0.024 | 0.872 |  |
| gamma-glutamylthreonine | Peptide | Gamma-glutamyl Amino Acid | 0.515 | 0.011 | 1.763 | 0.035 | 0.909 |  |
| gamma-glutamylvaline | Peptide | Gamma-glutamyl Amino Acid | 0.536 | 0.011 | 1.537 |  | 0.824 |  |
| gamma-glutamylleucine | Peptide | Gamma-glutamyl Amino Acid | 0.531 | 0.021 | 1.398 |  | 0.743 |  |
| gamma-glutamyltryptophan | Peptide | Gamma-glutamyl Amino Acid | 0.497 | 0.022 | 1.720 |  | 0.854 |  |
| gamma-glutamylalanine | Peptide | Gamma-glutamyl Amino Acid | 0.631 | 0.028 | 1.092 |  | 0.689 |  |
| gamma-glutamylisoleucine | Peptide | Gamma-glutamyl Amino Acid | 0.602 | 0.030 | 1.309 |  | 0.788 |  |
| gamma-glutamyl-2-aminobutyrate | Peptide | Gamma-glutamyl Amino Acid | 0.287 | 0.039 | 3.008 |  | 0.865 |  |
| gamma-glutamyltyrosine | Peptide | Gamma-glutamyl Amino Acid | 0.599 | 0.046 | 1.293 |  | 0.774 |  |
| erythronate | Carbohydrate | Aminosugar Metabolism | 0.746 | 0.039 | 0.979 |  | 0.730 | 0.028 |
| galactonate | Carbohydrate | Fructose, Mannose and Galactose Metabolism | 0.548 | 0.001 | 1.233 |  | 0.675 | 0.009 |
| 1,5-anhydroglucitol (1,5-AG) | Carbohydrate | Glycolysis, Gluconeogenesis, and Pyruvate Metabolism | 0.471 | < 0.001 | 0.987 |  | 0.465 | < 0.001 |
| arabitol | Carbohydrate | Pentose Metabolism | 0.548 | < 0.001 | 1.587 | 0.004 | 0.869 |  |
| threitol | Carbohydrate | Pentose Metabolism | 0.596 | 0.014 | 1.469 |  | 0.875 |  |
| xylonate | Carbohydrate | Pentose Metabolism | 0.642 | 0.017 | 0.926 |  | 0.595 | 0.008 |
| arabinose | Carbohydrate | Pentose Metabolism | 1.144 |  | 0.657 | 0.034 | 0.751 |  |
| tricarballylate | Energy | TCA Cycle | 0.153 | 0.007 | 1.872 |  | 0.286 | 0.021 |
| fumarate | Energy | TCA Cycle | 0.487 | 0.024 | 1.260 |  | 0.613 |  |
| malate | Energy | TCA Cycle | 0.521 | 0.036 | 1.231 |  | 0.642 |  |
| alpha-ketoglutarate | Energy | TCA Cycle | 0.633 | 0.043 | 1.251 |  | 0.792 |  |
| N-oleoyltaurine | Lipid | Endocannabinoid | 3.617 | 0.004 | 0.381 | 0.010 | 1.378 |  |
| oleic ethanolamide | Lipid | Endocannabinoid | 2.759 | 0.027 | 0.561 |  | 1.547 |  |
| propionylglycine | Lipid | Fatty Acid Metabolism (also BCAA Metabolism) | 0.497 | 0.022 | 1.484 |  | 0.737 |  |
| decanoylcarnitine | Lipid | Fatty Acid Metabolism(Acyl Carnitine) | 3.150 | < 0.001 | 0.571 | 0.006 | 1.798 |  |
| myristoleoylcarnitine | Lipid | Fatty Acid Metabolism(Acyl Carnitine) | 2.880 | 0.001 | 0.522 | 0.007 | 1.503 |  |
| laurylcarnitine | Lipid | Fatty Acid Metabolism(Acyl Carnitine) | 2.318 | 0.003 | 0.578 | 0.019 | 1.341 |  |
| oleoylcarnitine | Lipid | Fatty Acid Metabolism(Acyl Carnitine) | 2.192 | 0.005 | 0.633 | 0.047 | 1.388 |  |
| myristoylcarnitine | Lipid | Fatty Acid Metabolism(Acyl Carnitine) | 2.124 | 0.017 | 0.635 |  | 1.350 |  |
| heptanoyl glycine | Lipid | Fatty Acid Metabolism(Acyl Glycine) | 2.244 | 0.027 | 0.964 |  | 2.164 | 0.038 |
| hexanoylglycine | Lipid | Fatty Acid Metabolism(Acyl Glycine) | 2.008 | 0.044 | 0.338 | 0.010 | 0.678 |  |
| malonate (propanedioate) | Lipid | Fatty Acid Synthesis | 0.556 | 0.003 | 1.129 |  | 0.627 | 0.009 |
| 2-aminooctanoate | Lipid | Fatty Acid, Amino | 1.660 | 0.016 | 0.826 |  | 1.371 |  |
| 15-methylpalmitate (isobar with 2-methylpalmitate) | Lipid | Fatty Acid, Branched | 1.623 |  | 1.010 |  | 1.640 | 0.025 |
| hexadecanedioate | Lipid | Fatty Acid, Dicarboxylate | 1.531 | 0.004 | 0.759 | 0.039 | 1.162 |  |
| 2-hydroxyadipate | Lipid | Fatty Acid, Dicarboxylate | 0.376 | 0.005 | 1.826 |  | 0.686 |  |
| undecanedioate | Lipid | Fatty Acid, Dicarboxylate | 2.514 | 0.006 | 0.930 |  | 2.339 | 0.012 |
| azelate (nonanedioate) | Lipid | Fatty Acid, Dicarboxylate | 2.410 | 0.016 | 0.836 |  | 2.013 |  |
| tetradecanedioate | Lipid | Fatty Acid, Dicarboxylate | 1.413 | 0.024 | 0.880 |  | 1.243 |  |
| dimethylmalonic acid | Lipid | Fatty Acid, Dicarboxylate | 0.669 | 0.050 | 1.612 | 0.023 | 1.079 |  |
| 2-hydroxypalmitate | Lipid | Fatty Acid, Monohydroxy | 1.405 | 0.032 | 0.707 | 0.029 | 0.994 |  |
| chiro-inositol | Lipid | Inositol Metabolism | 0.419 | 0.001 | 1.585 |  | 0.665 | 0.043 |
| myo-inositol | Lipid | Inositol Metabolism | 0.700 | 0.048 | 1.153 |  | 0.807 |  |
| eicosenoate (20:1n9 or 11) | Lipid | Long Chain Fatty Acid | 2.826 | 0.004 | 0.512 | 0.022 | 1.448 |  |
| margarate (17:0) | Lipid | Long Chain Fatty Acid | 1.765 | 0.013 | 0.785 |  | 1.386 |  |
| stearate (18:0) | Lipid | Long Chain Fatty Acid | 1.388 | 0.044 | 0.917 |  | 1.272 |  |
| 1-pentadecanoylglycerophosphocholine (15:0) | Lipid | Lysolipid | 0.618 | 0.001 | 1.107 |  | 0.685 | 0.003 |
| 1-eicosapentaenoylglycerophosphocholine (20:5n3) | Lipid | Lysolipid | 0.221 | 0.004 | 0.950 |  | 0.210 | 0.004 |
| 1-docosapentaenoylglycerophosphocholine (22:5n6) | Lipid | Lysolipid | 4.360 | 0.004 | 0.821 |  | 3.578 | 0.024 |
| oleoyl-linoleoyl-glycerophosphocholine (2) | Lipid | Lysolipid | 1.365 | 0.006 | 1.039 |  | 1.418 | 0.002 |
| stearoyl-arachidonoyl-glycerophosphocholine (2) | Lipid | Lysolipid | 1.642 | 0.009 | 0.931 |  | 1.529 | 0.027 |
| 1-arachidonoylglyercophosphate | Lipid | Lysolipid | 2.647 | 0.011 | 0.565 |  | 1.496 |  |
| stearoyl-arachidonoyl-glycerophosphocholine (1) | Lipid | Lysolipid | 1.836 | 0.021 | 1.030 |  | 1.891 | 0.015 |
| 1-arachidonoylglycerophosphoinositol | Lipid | Lysolipid | 1.948 | 0.027 | 0.793 |  | 1.545 |  |
| 2-oleoylglycerophosphocholine | Lipid | Lysolipid | 1.627 | 0.028 | 0.825 |  | 1.343 |  |
| 1-arachidonoylglycerophosphocholine (20:4n6) | Lipid | Lysolipid | 1.417 | 0.037 | 1.023 |  | 1.449 | 0.025 |
| palmitoyl-arachidonoyl-glycerophosphocholine (1) | Lipid | Lysolipid | 1.421 | 0.038 | 1.166 |  | 1.656 | 0.002 |
| stearoyl-linoleoyl-glycerophosphocholine (2) | Lipid | Lysolipid | 1.275 | 0.044 | 1.090 |  | 1.390 | 0.006 |
| palmitoyl-palmitoyl-glycerophosphocholine (1) | Lipid | Lysolipid | 1.332 |  | 1.505 |  | 2.005 | 0.009 |
| 1-docosapentaenoylglycerophosphocholine (22:5n3) | Lipid | Lysolipid | 0.618 |  | 0.895 |  | 0.553 | 0.027 |
| palmitoyl-arachidonoyl-glycerophosphocholine (2) | Lipid | Lysolipid | 1.244 |  | 1.090 |  | 1.357 | 0.032 |
| palmitoyl-oleoyl-glycerophosphocholine (1) | Lipid | Lysolipid | 1.384 |  | 1.022 |  | 1.414 | 0.041 |
| 1-eicosatrienoylglycerophosphoethanolamine | Lipid | Lysolipid | 2.514 |  | 0.253 | 0.024 | 0.635 |  |
| pelargonate (9:0) | Lipid | Medium Chain Fatty Acid | 1.445 | 0.033 | 0.774 |  | 1.118 |  |
| 3-hydroxy-3-methylglutarate | Lipid | Mevalonate Metabolism | 0.587 | 0.018 | 1.465 |  | 0.861 |  |
| 1-docosahexaenoylglycerol | Lipid | Monoacylglycerol | 0.619 |  | 0.543 |  | 0.336 | 0.010 |
| glycerophosphorylcholine (GPC) | Lipid | Phospholipid Metabolism | 1.760 | 0.002 | 0.733 | 0.040 | 1.289 |  |
| choline | Lipid | Phospholipid Metabolism | 0.887 |  | 0.890 |  | 0.789 | 0.027 |
| ethanolamine | Lipid | Phospholipid Metabolism | 0.698 |  | 0.373 |  | 0.260 | 0.044 |
| eicosapentaenoate (EPA; 20:5n3) | Lipid | Polyunsaturated Fatty Acid (n3 and n6) | 0.303 | 0.001 | 0.823 |  | 0.250 | < 0.001 |
| mead acid (20:3n9) | Lipid | Polyunsaturated Fatty Acid (n3 and n6) | 4.432 | 0.002 | 0.618 |  | 2.740 |  |
| dihomo-linoleate (20:2n6) | Lipid | Polyunsaturated Fatty Acid (n3 and n6) | 4.007 | 0.004 | 0.460 | 0.030 | 1.842 |  |
| dihomo-linolenate (20:3n3 or n6) | Lipid | Polyunsaturated Fatty Acid (n3 and n6) | 1.985 | 0.005 | 0.630 | 0.029 | 1.250 |  |
| docosatrienoate (22:3n3) | Lipid | Polyunsaturated Fatty Acid (n3 and n6) | 2.643 | 0.020 | 0.318 | 0.012 | 0.841 |  |
| adrenate (22:4n6) | Lipid | Polyunsaturated Fatty Acid (n3 and n6) | 3.410 | 0.036 | 0.698 |  | 2.380 |  |
| arachidonate (20:4n6) | Lipid | Polyunsaturated Fatty Acid (n3 and n6) | 1.665 | 0.046 | 0.931 |  | 1.551 |  |
| docosadienoate (22:2n6) | Lipid | Polyunsaturated Fatty Acid (n3 and n6) | 1.798 | 0.049 | 0.569 |  | 1.023 |  |
| docosapentaenoate (n6 DPA; 22:5n6) | Lipid | Polyunsaturated Fatty Acid (n3 and n6) | 5.110 | < 0.001 | 0.463 | 0.002 | 2.366 |  |
| inosine | Nucleotide | Purine Metabolism, (Hypo)Xanthine/Inosine containing | 5.644 |  | 0.019 | 0.041 | 0.107 |  |
| N6-carbamoylthreonyladenosine | Nucleotide | Purine Metabolism, Adenine containing | 1.627 | 0.037 | 0.581 | 0.024 | 0.946 |  |
| N1-methylguanosine | Nucleotide | Purine Metabolism, Guanine containing | 1.402 | 0.016 | 0.664 | 0.006 | 0.931 |  |
| N2,N2-dimethylguanosine | Nucleotide | Purine Metabolism, Guanine containing | 1.656 | 0.046 | 0.741 |  | 1.228 |  |
| guanosine | Nucleotide | Purine Metabolism, Guanine containing | 2.306 |  | 0.422 | 0.048 | 0.973 |  |
| 3-ureidopropionate | Nucleotide | Pyrimidine Metabolism, Uracil containing | 0.585 | 0.005 | 1.200 |  | 0.702 | 0.035 |
| 5-methyluridine (ribothymidine) | Nucleotide | Pyrimidine Metabolism, Uracil containing | 2.153 | 0.031 | 0.722 |  | 1.554 |  |
| beta-alanine | Nucleotide | Pyrimidine Metabolism, Uracil containing | 0.596 |  | 0.641 |  | 0.382 | 0.009 |
| glucarate (saccharate) | Cofactors and Vitamins | Ascorbate and Aldarate Metabolism | 0.615 | 0.014 | 1.424 |  | 0.876 |  |
| gulonic acid | Cofactors and Vitamins | Ascorbate and Aldarate Metabolism | 0.520 | 0.034 | 1.521 |  | 0.790 |  |
| biliverdin | Cofactors and Vitamins | Hemoglobin and Porphyrin Metabolism | 1.803 |  | 0.419 | 0.015 | 0.755 |  |
| nicotinamide N-oxide | Cofactors and Vitamins | Nicotinate and Nicotinamide Metabolism | 2.599 | 0.030 | 0.699 |  | 1.816 |  |
| 1-methylnicotinamide | Cofactors and Vitamins | Nicotinate and Nicotinamide Metabolism | 2.835 | 0.047 | 0.354 | 0.047 | 1.004 |  |
| riboflavin (Vitamin B2) | Cofactors and Vitamins | Riboflavin Metabolism | 0.589 | 0.003 | 1.347 |  | 0.793 |  |
| flavin adenine dinucleotide (FAD) | Cofactors and Vitamins | Riboflavin Metabolism | 0.834 | 0.042 | 1.016 |  | 0.848 |  |
| dihydrobiopterin | Cofactors and Vitamins | Tetrahydrobiopterin Metabolism | 0.750 |  | 0.673 |  | 0.505 | 0.008 |
| 4-sulfooxy-methylbenzoate | Xenobiotics | Benzoate Metabolism | 0.158 | 0.010 | 4.081 |  | 0.644 |  |
| 4-hydroxyhippurate | Xenobiotics | Benzoate Metabolism | 0.359 | 0.016 | 1.519 |  | 0.546 |  |
| 3-methoxycatechol sulfate (2) | Xenobiotics | Benzoate Metabolism | 0.445 | 0.026 | 0.804 |  | 0.358 | 0.011 |
| p-hydroxybenzaldehyde | Xenobiotics | Benzoate Metabolism | 0.668 | 0.042 | 1.115 |  | 0.745 |  |
| 2-hydroxyhippurate (salicylurate) | Xenobiotics | Benzoate Metabolism | 0.056 | < 0.001 | 2.100 |  | 0.118 | < 0.001 |
| 3-methyl catechol sulfate (1) | Xenobiotics | Benzoate Metabolism | 0.075 | < 0.001 | 0.764 |  | 0.057 | < 0.001 |
| S-(3-hydroxypropyl)mercapturic acid (HPMA) | Xenobiotics | Chemical | 0.354 | < 0.001 | 1.285 |  | 0.454 | 0.001 |
| 3-hydroxypyridine sulfate | Xenobiotics | Chemical | 0.094 | 0.001 | 0.766 |  | 0.072 | 0.001 |
| 2-oxo-1-pyrrolidinepropionate | Xenobiotics | Chemical | 0.366 | 0.001 | 1.081 |  | 0.396 | 0.001 |
| 2-hydroxyisobutyrate | Xenobiotics | Chemical | 1.507 | 0.034 | 0.974 |  | 1.468 |  |
| N-methylpipecolate | Xenobiotics | Chemical | 0.230 | < 0.001 | 1.820 | 0.032 | 0.418 | < 0.001 |
| 4-hydroxychlorothalonil | Xenobiotics | Chemical | 0.792 |  | 2.298 | < 0.001 | 1.819 | 0.002 |
| glycolate (hydroxyacetate) | Xenobiotics | Chemical | 0.862 |  | 0.992 | 0.013 | 0.855 | 0.017 |
| 6-oxopiperidine-2-carboxylic acid | Xenobiotics | Drug | 0.563 | 0.001 | 1.353 |  | 0.762 |  |
| 4-acetylphenol sulfate | Xenobiotics | Drug | 0.355 | 0.006 | 1.420 |  | 0.504 | 0.030 |
| salicylate | Xenobiotics | Drug | 0.079 | < 0.001 | 2.089 |  | 0.165 | < 0.001 |
| 2-piperidinone | Xenobiotics | Food Component/  Plant | 0.475 | 0.015 | 1.621 |  | 0.770 |  |
| N-(2-furoyl)glycine | Xenobiotics | Food Component/  Plant | 0.248 | 0.037 | 1.662 |  | 0.412 |  |
| daidzein | Xenobiotics | Food Component/  Plant | 0.363 | 0.043 | 2.614 |  | 0.948 |  |
| homostachydrine | Xenobiotics | Food Component/  Plant | 0.083 | < 0.001 | 1.172 |  | 0.097 | < 0.001 |
| methyl glucopyranoside (alpha + beta) | Xenobiotics | Food Component/  Plant | 0.019 | < 0.001 | 1.255 |  | 0.024 | < 0.001 |
| quinate | Xenobiotics | Food Component/  Plant | 0.029 | < 0.001 | 1.987 |  | 0.059 | < 0.001 |
| stachydrine | Xenobiotics | Food Component/  Plant | 0.095 | < 0.001 | 1.378 |  | 0.131 | < 0.001 |
| 2,3-dihydroxyisovalerate | Xenobiotics | Food Component/  Plant | 0.522 |  | 4.385 | < 0.001 | 2.288 | < 0.001 |
