# Supplementary material for: A comprehensive multiomics approach toward understanding the relationship between aging and dementia
Source: Aging (Albany NY). 2015 Nov 11;7(11):937–52. doi: 10.18632/aging.100838 (PMC4694064; doi:10.18632/aging.100838)
Supplement: Supplementary file 3 [file aging-07-0937-s003.docx]

| **Supplemental Table 2. Altered biochemicals in the cortex** | | | | | | | | |
| --- | --- | --- | --- | --- | --- | --- | --- | --- |
|  |  |  | **Old / Young** | | **J147 / Old** | | **J147 / Young** | |
| **BIOCHEMICAL** | **SUPER PATHWAY** | **SUB PATHWAY** | Fold change | p value | Fold change | p value | Fold change | p value |
| asparagine | Amino Acid | Alanine and Aspartate Metabolism | 0.840 | 0.026 | 1.110 |  | 0.933 |  |
| glutamate | Amino Acid | Glutamate Metabolism | 0.862 | 0.001 | 1.105 | 0.015 | 0.953 |  |
| pyroglutamine | Amino Acid | Glutamate Metabolism | 1.199 | 0.002 | 1.051 |  | 1.261 | < 0.001 |
| glutamine | Amino Acid | Glutamate Metabolism | 1.188 | 0.008 | 0.955 |  | 1.134 |  |
| N-acetylglutamate | Amino Acid | Glutamate Metabolism | 0.855 | 0.009 | 1.084 |  | 0.927 |  |
| N-acetyl-aspartyl-glutamate (NAAG) | Amino Acid | Glutamate Metabolism | 0.762 | 0.021 | 1.040 |  | 0.792 | 0.043 |
| glutathione, oxidized (GSSG) | Amino Acid | Glutathione Metabolism | 0.859 | 0.019 | 0.973 |  | 0.836 | 0.007 |
| cysteine-glutathione disulfide | Amino Acid | Glutathione Metabolism | 0.932 |  | 0.704 |  | 0.656 | 0.042 |
| N-acetylserine | Amino Acid | Glycine, Serine and Threonine Metabolism | 0.856 | < 0.001 | 0.920 |  | 0.787 | < 0.001 |
| serine | Amino Acid | Glycine, Serine and Threonine Metabolism | 0.777 | 0.010 | 0.998 |  | 0.775 | 0.009 |
| threonine | Amino Acid | Glycine, Serine and Threonine Metabolism | 0.838 | 0.017 | 1.064 |  | 0.891 |  |
| allo-threonine | Amino Acid | Glycine, Serine and Threonine Metabolism | 0.803 | 0.043 | 0.976 |  | 0.784 | 0.027 |
| histidine | Amino Acid | Histidine Metabolism | 1.364 | 0.001 | 0.945 |  | 1.288 | 0.003 |
| trans-urocanate | Amino Acid | Histidine Metabolism | 2.075 | 0.005 | 0.676 |  | 1.402 |  |
| 3-hydroxyisobutyrate | Amino Acid | Leucine, Isoleucine and Valine Metabolism | 0.722 | 0.001 | 1.036 |  | 0.748 | 0.001 |
| ethylmalonate | Amino Acid | Leucine, Isoleucine and Valine Metabolism | 1.336 | 0.025 | 0.768 | 0.038 | 1.025 |  |
| methylsuccinate | Amino Acid | Leucine, Isoleucine and Valine Metabolism | 1.119 | 0.027 | 0.890 | 0.023 | 0.996 |  |
| N-acetylleucine | Amino Acid | Leucine, Isoleucine and Valine Metabolism | 0.838 |  | 0.918 | 0.019 | 0.769 |  |
| saccharopine | Amino Acid | Lysine Metabolism | 0.574 | 0.001 | 1.362 |  | 0.782 |  |
| pipecolate | Amino Acid | Lysine Metabolism | 0.784 | 0.030 | 0.888 |  | 0.696 | 0.004 |
| 2-hydroxybutyrate (AHB) | Amino Acid | Methionine, Cysteine, SAM and Taurine Metabolism | 0.603 | < 0.001 | 1.372 | 0.013 | 0.827 |  |
| methionine | Amino Acid | Methionine, Cysteine, SAM and Taurine Metabolism | 1.871 | 0.001 | 0.798 |  | 1.494 | 0.034 |
| S-adenosylmethionine (SAM) | Amino Acid | Methionine, Cysteine, SAM and Taurine Metabolism | 0.820 | 0.013 | 1.148 |  | 0.941 |  |
| S-methylcysteine | Amino Acid | Methionine, Cysteine, SAM and Taurine Metabolism | 1.931 | 0.023 | 0.866 |  | 1.672 |  |
| N-acetyltaurine | Amino Acid | Methionine, Cysteine, SAM and Taurine Metabolism | 0.829 | 0.028 | 0.920 |  | 0.763 | 0.004 |
| 3-[3-(sulfooxy)phenyl]propanoic acid | Amino Acid | Phenylalanine and Tyrosine Metabolism | 10.441 | 0.006 | 0.404 |  | 4.221 |  |
| phenylalanine | Amino Acid | Phenylalanine and Tyrosine Metabolism | 1.163 | 0.031 | 0.924 |  | 1.074 |  |
| N-acetyltyrosine | Amino Acid | Phenylalanine and Tyrosine Metabolism | 0.885 |  | 0.735 |  | 0.651 | 0.017 |
| indoleacetate | Amino Acid | Tryptophan Metabolism | 3.847 | 0.006 | 0.696 |  | 2.676 |  |
| pro-hydroxy-pro | Amino Acid | Urea cycle; Arginine and Proline Metabolism | 0.489 | < 0.001 | 1.068 |  | 0.522 | < 0.001 |
| ornithine | Amino Acid | Urea cycle; Arginine and Proline Metabolism | 0.496 | 0.010 | 0.715 |  | 0.354 | 0.002 |
| trans-4-hydroxyproline | Amino Acid | Urea cycle; Arginine and Proline Metabolism | 0.239 | < 0.001 | 1.536 |  | 0.367 | < 0.001 |
| homocitrulline | Amino Acid | Urea cycle; Arginine and Proline Metabolism | 0.495 | 0.002 | 0.975 | 0.003 | 0.482 |  |
| citrulline | Amino Acid | Urea cycle; Arginine and Proline Metabolism | 1.168 |  | 1.379 | 0.048 | 1.611 | 0.008 |
| alanylalanine | Peptide | Dipeptide | 0.440 | 0.040 | 0.849 |  | 0.373 | 0.022 |
| pyroglutamylvaline | Peptide | Dipeptide | 1.199 |  | 0.521 | 0.024 | 0.625 |  |
| homocarnosine | Peptide | Dipeptide Derivative | 1.676 | 0.007 | 0.877 |  | 1.471 |  |
| gamma-glutamylglutamine | Peptide | Gamma-glutamyl Amino Acid | 1.443 | 0.002 | 0.950 |  | 1.371 | 0.006 |
| gamma-glutamylmethionine | Peptide | Gamma-glutamyl Amino Acid | 2.292 | 0.002 | 0.762 |  | 1.746 |  |
| gamma-glutamylphenylalanine | Peptide | Gamma-glutamyl Amino Acid | 1.442 | 0.005 | 1.006 |  | 1.450 | 0.006 |
| N-acetylneuraminate | Carbohydrate | Aminosugar Metabolism | 0.887 | 0.006 | 0.983 |  | 0.872 | 0.002 |
| erythronate | Carbohydrate | Aminosugar Metabolism | 0.915 | 0.032 | 1.034 |  | 0.946 |  |
| N-acetylglucosamine 6-phosphate | Carbohydrate | Aminosugar Metabolism | 0.906 |  | 0.965 |  | 0.874 | 0.039 |
| pyruvate | Carbohydrate | Glycolysis, Gluconeogenesis, and Pyruvate Metabolism | 0.671 | 0.001 | 1.083 |  | 0.727 | 0.005 |
| 3-phosphoglycerate | Carbohydrate | Glycolysis, Gluconeogenesis, and Pyruvate Metabolism | 1.741 | 0.016 | 0.732 |  | 1.275 |  |
| phosphoenolpyruvate (PEP) | Carbohydrate | Glycolysis, Gluconeogenesis, and Pyruvate Metabolism | 1.661 | 0.020 | 0.804 |  | 1.336 |  |
| 2-phosphoglycerate | Carbohydrate | Glycolysis, Gluconeogenesis, and Pyruvate Metabolism | 1.568 | 0.049 | 0.866 |  | 1.357 |  |
| 1,5-anhydroglucitol (1,5-AG) | Carbohydrate | Glycolysis, Gluconeogenesis, and Pyruvate Metabolism | 0.471 | < 0.001 | 0.876 |  | 0.412 | < 0.001 |
| UDP-glucuronate | Carbohydrate | Nucleotide Sugar | 0.819 | 0.007 | 1.030 |  | 0.844 | 0.018 |
| UDP-N-acetylglucosamine | Carbohydrate | Nucleotide Sugar | 0.848 | 0.008 | 1.011 |  | 0.858 | 0.013 |
| cytidine 5'-monophospho-N-acetylneuraminic acid | Carbohydrate | Nucleotide Sugar | 0.892 | 0.044 | 1.015 |  | 0.905 |  |
| arabinose | Carbohydrate | Pentose Metabolism | 1.432 | 0.001 | 0.913 |  | 1.307 | 0.011 |
| ribitol | Carbohydrate | Pentose Metabolism | 0.625 | < 0.001 | 1.319 | 0.012 | 0.824 | 0.026 |
| xylitol | Carbohydrate | Pentose Metabolism | 0.651 |  | 0.871 |  | 0.567 | 0.027 |
| arabonate/xylonate | Carbohydrate | Pentose Phosphate Pathway | 1.206 | 0.015 | 0.899 |  | 1.084 |  |
| sedoheptulose-7-phosphate | Carbohydrate | Pentose Phosphate Pathway | 0.782 | 0.032 | 1.056 |  | 0.826 |  |
| ribose 1-phosphate | Carbohydrate | Pentose Phosphate Pathway | 1.004 |  | 0.840 | 0.045 | 0.843 |  |
| alpha-ketoglutarate | Energy | TCA Cycle | 0.895 | 0.019 | 1.109 | 0.028 | 0.993 |  |
| succinylcarnitine | Energy | TCA Cycle | 1.362 | 0.001 | 0.829 | 0.021 | 1.130 |  |
| citrate | Energy | TCA Cycle | 1.262 | 0.033 | 1.181 |  | 1.409 | 0.002 |
| isocitrate | Energy | TCA Cycle | 1.637 | 0.037 | 1.049 |  | 1.717 | 0.020 |
| deoxycarnitine | Lipid | Carnitine Metabolism | 0.914 |  | 0.967 |  | 0.883 | 0.025 |
| acetyl CoA | Lipid | Fatty Acid Metabolism | 1.608 | 0.008 | 0.930 |  | 1.495 | 0.027 |
| propionylglycine | Lipid | Fatty Acid Metabolism (also BCAA Metabolism) | 0.589 |  | 0.880 |  | 0.518 | 0.041 |
| acetylcarnitine | Lipid | Fatty Acid Metabolism(Acyl Carnitine) | 1.225 | 0.016 | 0.910 |  | 1.114 |  |
| malonylcarnitine | Lipid | Fatty Acid Synthesis | 1.134 |  | 1.285 | 0.018 | 1.456 | 0.002 |
| 2-methylmalonyl carnitine | Lipid | Fatty Acid Synthesis | 0.847 |  | 0.798 |  | 0.676 | 0.043 |
| 2-hydroxyadipate | Lipid | Fatty Acid, Dicarboxylate | 0.579 | 0.001 | 1.137 |  | 0.658 | 0.006 |
| 2-hydroxyglutarate | Lipid | Fatty Acid, Dicarboxylate | 0.838 | 0.016 | 0.980 |  | 0.821 | 0.009 |
| 2-hydroxypalmitate | Lipid | Fatty Acid, Monohydroxy | 1.839 | 0.001 | 1.028 |  | 1.891 | < 0.001 |
| 3-hydroxylaurate | Lipid | Fatty Acid, Monohydroxy | 1.439 | 0.019 | 0.821 |  | 1.181 |  |
| 2-hydroxystearate | Lipid | Fatty Acid, Monohydroxy | 1.725 | < 0.001 | 1.117 |  | 1.927 | < 0.001 |
| scyllo-inositol | Lipid | Inositol Metabolism | 0.783 |  | 0.923 |  | 0.723 | 0.016 |
| palmitoyl-oleoyl-glycerophosphoglycerol (2) | Lipid | Lysolipid | 0.934 | 0.019 | 0.983 |  | 0.918 | 0.005 |
| stearoyl-arachidonoyl-glycerophosphoserine (1) | Lipid | Lysolipid | 1.186 |  | 1.047 |  | 1.241 | 0.040 |
| 3-hydroxy-3-methylglutarate | Lipid | Mevalonate Metabolism | 0.824 | < 0.001 | 1.002 |  | 0.826 | < 0.001 |
| 1-stearoylglycerol (1-monostearin) | Lipid | Monoacylglycerol | 0.733 | 0.007 | 1.085 |  | 0.796 | 0.033 |
| ethanolamine | Lipid | Phospholipid Metabolism | 0.841 | 0.016 | 1.046 |  | 0.880 |  |
| cytidine 5'-diphosphocholine | Lipid | Phospholipid Metabolism | 0.856 | 0.029 | 0.991 |  | 0.849 | 0.022 |
| cytidine-5'-diphosphoethanolamine | Lipid | Phospholipid Metabolism | 0.899 | 0.030 | 1.035 |  | 0.931 |  |
| choline phosphate | Lipid | Phospholipid Metabolism | 0.954 |  | 0.946 |  | 0.903 | 0.037 |
| docosapentaenoate (n3 DPA; 22:5n3) | Lipid | Polyunsaturated Fatty Acid (n3 and n6) | 0.592 | < 0.001 | 1.085 |  | 0.642 | 0.001 |
| eicosapentaenoate (EPA; 20:5n3) | Lipid | Polyunsaturated Fatty Acid (n3 and n6) | 0.425 | 0.005 | 0.672 |  | 0.286 | 0.001 |
| dihomo-linolenate (20:3n3 or n6) | Lipid | Polyunsaturated Fatty Acid (n3 and n6) | 0.636 | < 0.0001 | 1.277 | 0.004 | 0.812 | 0.010 |
| docosapentaenoate (n6 DPA; 22:5n6) | Lipid | Polyunsaturated Fatty Acid (n3 and n6) | 1.916 | 0.034 | 1.157 |  | 2.217 | 0.006 |
| docosatrienoate (22:3n3) | Lipid | Polyunsaturated Fatty Acid (n3 and n6) | 0.519 | 0.037 | 1.180 |  | 0.612 |  |
| cholestanol | Lipid | Sterol | 1.510 | 0.025 | 0.907 |  | 1.369 |  |
| urate | Nucleotide | Purine Metabolism, (Hypo)Xanthine/  Inosine containing | 1.382 | 0.017 | 0.859 |  | 1.186 |  |
| adenosine 3',5'-cyclic monophosphate (cAMP) | Nucleotide | Purine Metabolism, Adenine containing | 1.218 | 0.031 | 0.813 | 0.024 | 0.990 |  |
| adenylosuccinate | Nucleotide | Purine Metabolism, Adenine containing | 1.101 |  | 0.166 | 0.024 | 0.183 | 0.043 |
| adenosine 5'-monophosphate (AMP) | Nucleotide | Purine Metabolism, Adenine containing | 1.122 |  | 0.617 | 0.043 | 0.692 |  |
| orotidine | Nucleotide | Pyrimidine Metabolism, Orotate containing | 0.894 | 0.035 | 1.042 |  | 0.932 |  |
| N-acetyl-beta-alanine | Nucleotide | Pyrimidine Metabolism, Uracil containing | 1.129 |  | 0.698 | 0.016 | 0.788 |  |
| ascorbate (Vitamin C) | Cofactors and Vitamins | Ascorbate and Aldarate Metabolism | 1.198 |  | 1.030 |  | 1.234 | 0.047 |
| nicotinamide ribonucleotide (NMN) | Cofactors and Vitamins | Nicotinate and Nicotinamide Metabolism | 1.269 |  | 1.062 |  | 1.348 | 0.043 |
| pantothenate | Cofactors and Vitamins | Pantothenate and CoA Metabolism | 1.735 | 0.001 | 0.828 |  | 1.437 | 0.020 |
| 3'-dephosphocoenzyme A | Cofactors and Vitamins | Pantothenate and CoA Metabolism | 1.419 | 0.034 | 1.097 |  | 1.557 | 0.006 |
| phosphopantetheine | Cofactors and Vitamins | Pantothenate and CoA Metabolism | 1.557 | 0.036 | 1.203 |  | 1.874 | 0.002 |
| dihydrobiopterin | Cofactors and Vitamins | Tetrahydrobiopterin Metabolism | 0.678 |  | 0.904 |  | 0.613 | 0.031 |
| salicylate | Xenobiotics | Drug | 0.604 | 0.034 | 1.081 |  | 0.653 |  |
| ergothioneine | Xenobiotics | Food Component/Plant | 2.109 | < 0.001 | 0.631 | 0.003 | 1.331 |  |
| 2,3-dihydroxyisovalerate | Xenobiotics | Food Component/Plant | 2.057 | 0.023 | 0.510 | 0.029 | 1.050 |  |
| methyl glucopyranoside (alpha + beta) | Xenobiotics | Food Component/Plant | 0.021 | < 0.001 | 1.552 |  | 0.033 | < 0.001 |
| stachydrine | Xenobiotics | Food Component/Plant | 0.165 | < 0.001 | 0.884 |  | 0.146 | < 0.001 |
| homocitrate | Xenobiotics | Food Component/Plant | 0.901 |  | 1.226 | 0.019 | 1.105 |  |
